# The zinc transporter ZIPT-7.1 regulates sperm activation in nematodes

**DOI:** 10.1371/journal.pbio.2005069

**Published:** 2018-06-07

**Authors:** Yanmei Zhao, Chieh-Hsiang Tan, Amber Krauchunas, Andrea Scharf, Nicholas Dietrich, Kurt Warnhoff, Zhiheng Yuan, Marina Druzhinina, Sam Guoping Gu, Long Miao, Andrew Singson, Ronald E. Ellis, Kerry Kornfeld

**Affiliations:** 1 Key Laboratory of RNA Biology, Institute of Biophysics, CAS Center for Excellence in Biomacromolecules, Chinese Academy of Sciences, Beijing, China; 2 College of Life Sciences, University of Chinese Academy of Sciences, Beijing, China; 3 Department of Molecular Biology, Rowan University SOM, Stratford, New Jersey, United States of America; 4 Department of Developmental Biology, Washington University School of Medicine, St. Louis, Missouri, United States of America; 5 Waksman Institute, Rutgers University, Piscataway, New Jersey, United States of America; 6 Department of Molecular Biology and Biochemistry, Rutgers University, Piscataway, New Jersey, United States of America; University of Minnesota, United States of America

## Abstract

Sperm activation is a fascinating example of cell differentiation, in which immotile spermatids undergo a rapid and dramatic transition to become mature, motile sperm. Because the sperm nucleus is transcriptionally silent, this transition does not involve transcriptional changes. Although *Caenorhabditis elegans* is a leading model for studies of sperm activation, the mechanisms by which signaling pathways induce this transformation remain poorly characterized. Here we show that a conserved transmembrane zinc transporter, ZIPT-7.1, regulates the induction of sperm activation in *Caenorhabditis* nematodes. The *zipt-7*.*1* mutant hermaphrodites cannot self-fertilize, and males reproduce poorly, because mutant spermatids are defective in responding to activating signals. The *zipt-7*.*1* gene is expressed in the germ line and functions in germ cells to promote sperm activation. When expressed in mammalian cells, ZIPT-7.1 mediates zinc transport with high specificity and is predominantly located on internal membranes. Finally, genetic epistasis places *zipt-7*.*1* at the end of the *spe-8* sperm activation pathway, and ZIPT-7.1 binds SPE-4, a presenilin that regulates sperm activation. Based on these results, we propose a new model for sperm activation. In spermatids, inactive ZIPT-7.1 is localized to the membranous organelles, which contain higher levels of zinc than the cytoplasm. When sperm activation is triggered, ZIPT-7.1 activity increases, releasing zinc from internal stores. The resulting increase in cytoplasmic zinc promotes the phenotypic changes characteristic of activation. Thus, zinc signaling is a key step in the signal transduction process that mediates sperm activation, and we have identified a zinc transporter that is central to this activation process.

## Author summary

Sperm are specialized cells with transcriptionally silent DNA that has been packaged for delivery into the egg. In their final step of development, immature sperm undergo a rapid transition from nonmotile cells to mature, motile sperm capable of fertilization. The signals that trigger this change are not clearly understood. By identifying mutants in the roundworm *Caenorhabditis elegans* that are defective in sperm activation, we discovered a conserved transmembrane protein, ZIPT-7.1, that transports zinc and promotes sperm activation in both sexes. ZIPT-7.1 is expressed in the germ line and functions there to control sperm activation. When expressed ectopically in mammalian cells, the protein specifically transports zinc across membranes and localizes primarily to membranes within the cell. Previous genetic studies had identified two pathways that mediate sperm activation in *C*. *elegans*, and our results suggest that *zipt-7*.*1* acts at the end of one of these two, the *spe-8* pathway. We propose that when this pathway triggers sperm activation, it acts through ZIPT-7.1, which mediates the release of zinc from internal stores in the immature sperm. This released zinc functions as a second messenger to promote the differentiation of mature, motile sperm.

## Introduction

In animals, each differentiated cell type expresses a unique set of genes, resulting in the characteristic array of proteins that together confer its identity. These proteins equip the cell for the specific functions it needs to perform its role within a complex animal. Sperm provide a fascinating example of how multiple specializations combine to promote one distinct function—delivering the male pronucleus to the egg to produce a fertilized zygote. One specialization is that sperm chromatin has been compacted to prepare for its delivery into the egg, resulting in transcriptional silencing [1, 2]. As a result, changes in sperm phenotype or behavior must be accomplished without new gene transcription. A second specialization is that inactive sperm from many species are stored until ejaculation, when they undergo dramatic postmeiotic transitions called sperm activation, capacitation, or spermiogenesis [3, 4]. In this process, the immotile spermatid becomes a mature, motile sperm. Precisely regulated activation conserves energy until it is needed to drive motility and fertilization.

Only the early stages of spermatogenesis involve changes in gene expression; thus, sperm activation occurs in cells with transcriptionally silent nuclei. Because well-established pathways such as wnt, hedgehog, receptor tyrosine kinase, TGFβ, and Notch do not appear to mediate activation, sperm are likely to use novel signaling pathways to induce this phenotype. And because sperm activation is widespread in animals, the mechanisms that control it might be ancient and conserved. To date, these mechanisms remain poorly understood.

The nematode *Caenorhabditis elegans* is ideal for studies of sperm differentiation and activation. In this species, inactive spermatids are round and immotile, with membranous organelles located just beneath the plasma membrane [5]. During activation, these organelles fuse with the plasma membrane, and major sperm protein (MSP) is reorganized into filaments that generate a pseudopod for crawling. This amoeboid movement is common in nematode sperm and may be adapted to the folded surfaces of the female or hermaphrodite reproductive tracts.

Forward genetic screens for sterile animals have resulted in the identification of many genes that are essential for one or more steps of sperm development in *C*. *elegans* [6, 7]. The molecular analysis of these genes has identified proteins that participate in two distinct signal transduction pathways that regulate sperm activation [4]. One of these pathways is triggered by a secreted trypsin protease [8], but the signal for the other pathway remains elusive. Although several chemicals that can activate sperm in vitro have been identified [9, 10], they have not resolved this problem. Here we demonstrate that the zinc transporter ZIPT-7.1 plays a critical role in this second sperm activation pathway.

To identify new genes involved in sperm biology, we analyzed mutations that cause hermaphrodites to become self-sterile. The new mutations *hc130* and *as42* cause a loss-of-function in *zipt-7*.*1*, resulting in partial or complete sterility in both sexes. This defect is due to a failure of sperm activation, a function conserved in the related nematode *C*. *tropicalis*. The ZIPT-7.1 protein is homologous to mammalian ZIP7, a zinc transporter from the ZIP family. We confirmed that *C*. *elegans* ZIPT-7.1 can act as a zinc-selective importer in cultured cells. Moreover, ZIPT-7.1 is expressed in the nematode germ line, consistent with a function in sperm. Genetic studies indicate that it interacts with other genes involved in sperm activation and functions downstream of *spe-6*. Finally, ZIPT-7.1 can bind the presenilin SPE-4.

Thus, we propose a new model for sperm activation. In spermatids, inactive ZIPT-7.1 is localized to the membranous organelles, which contain much higher levels of zinc than the cytoplasm. When activation is triggered, a signal transduced by the SPE-8 group of proteins opposes SPE-4 and SPE-6. As their function decreases, ZIPT-7.1 becomes active and transports zinc into the cytoplasm. The resulting increase in cytoplasmic zinc promotes the phenotypic changes that are characteristic of activation, including motility. Thus, the release of zinc from internal stores is a key part of the signal transduction process that mediates sperm activation.

These discoveries have important implications for the fields of zinc biology and sperm activation. Zinc is essential for all life and has well-established functions as a cofactor for numerous proteins. Zinc binding is necessary for the tertiary structure of many of these proteins, such as zinc finger transcription factors, and zinc binding to many enzymes is critical for catalysis. Although it has been suggested that changes in zinc concentration in specific compartments might have second messenger effects, it has been difficult to demonstrate this type of signaling. The best-established setting is the extracellular release of zinc during synaptic transmission, which changes the concentration of zinc in the synaptic cleft [11]. By contrast, examples of zinc signaling that control cell fate and development are lacking. Our demonstration that a zinc signal controls sperm activation places zinc signaling in a specific biological context, in which changes in cell identity cannot be mediated by changes in gene expression. Furthermore, we have identified a zinc transporter that is central to this activation process. Finally, our discoveries show that signal transduction using zinc can control how cells differentiate during development.

## Results

### Identification of the nematode *zipt-7*.*1* gene

**Fig 1:**
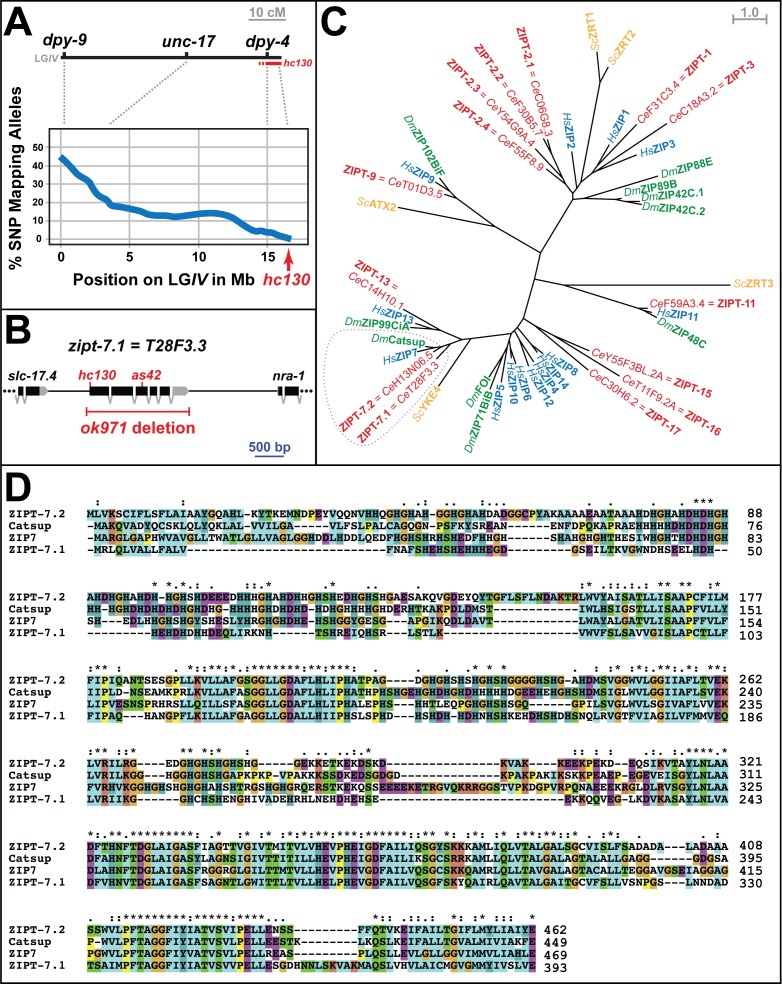
The *hc130* mutation alters *zipt-7*.*1*, which encodes a ZIP family transporter. (A) Genetic map of linkage group IV (upper) and a corresponding portion of the physical map (lower). Blue line indicates the frequency of CB4856 SNP alleles in homozygous *hc130* mutant animals, and red shows the inferred position of the *hc130* mutation. (B) Diagram of the physical map showing *zipt-7*.*1* gene structure and portions of flanking genes. Predicted exons are boxes, coding regions are black, and untranslated regions are gray. The extent of the *ok971* deletion mutation and the positions of *hc130* and *as42* are marked. (C) A maximum likelihood tree illustrating evolutionary relationships between predicted ZIP proteins from *Caenorhabditis elegans* (red), *Drosophila melanogaster* (green), *Homo sapiens* (blue), and *Saccharomyces cerevisia* (yellow). The ZIP7 family is circled. (D) An alignment of predicted ZIP7 proteins from *C*. *elegans* (ZIPT-7.1 and ZIPT-7.2), *D*. *melanogaster* (Catsup), and *H*. *sapiens* (ZIP7). Identical residues are marked “*” and similar ones “:”; chemical properties are indicated by color according to ClustalX conventions. The individual numerical values for panel A can be found in S1 Data.

Two independent lines of investigation converged on *zipt-7*.*1* as a critical regulator of fertility. The first approach was based on a forward genetic screen for sterile *C*. *elegans* hermaphrodites, which led to the identification of the recessive mutation *hc130*. Genetic mapping experiments were used to position this mutation to the right of *dpy-4*, near the end of chromosome *IV* (Fig 1A), and whole genome sequencing revealed a missense mutation that eliminated the ATG start codon of T28F3.3, a gene located in this region (Fig 1B).

The second approach was designed to elucidate mechanisms of zinc biology by conducting a reverse genetic study of *C*. *elegans* genes encoding ZIP proteins. Homology searches identified 14 such genes, and phylogenetic analyses revealed that many are closely related to human proteins. Thus, we named these genes ZRT- and IRT-like protein transporters (*zipt*) and assigned numbers corresponding to the most similar human genes (Fig 1C, S1 Table). By analyzing deletion alleles, we discovered that *zipt-7*.*1(ok971)*, which deletes T28F3.3, caused hermaphrodite sterility. Complementation tests showed that *hc130*/*ok971* heterozygotes were sterile, confirming that the missense mutation identified in T28F3.3 causes the *hc130* phenotype.

Finally, we used a screening procedure in which sterile mutants were identified by their failure to form “bags-of-worms” when prevented from laying eggs [12] to identify another mutation that causes this phenotype. This allele, *as42*, has a G797A mutation in T28F3.3, which changes a glycine to glutamic acid within a predicted transmembrane domain. Taken together, these three alleles identify a previously uncharacterized *zipt* gene required for nematode fertility.

### *zipt-7*.*1* is needed to promote sperm function in both hermaphrodites and males

**Fig 2:**
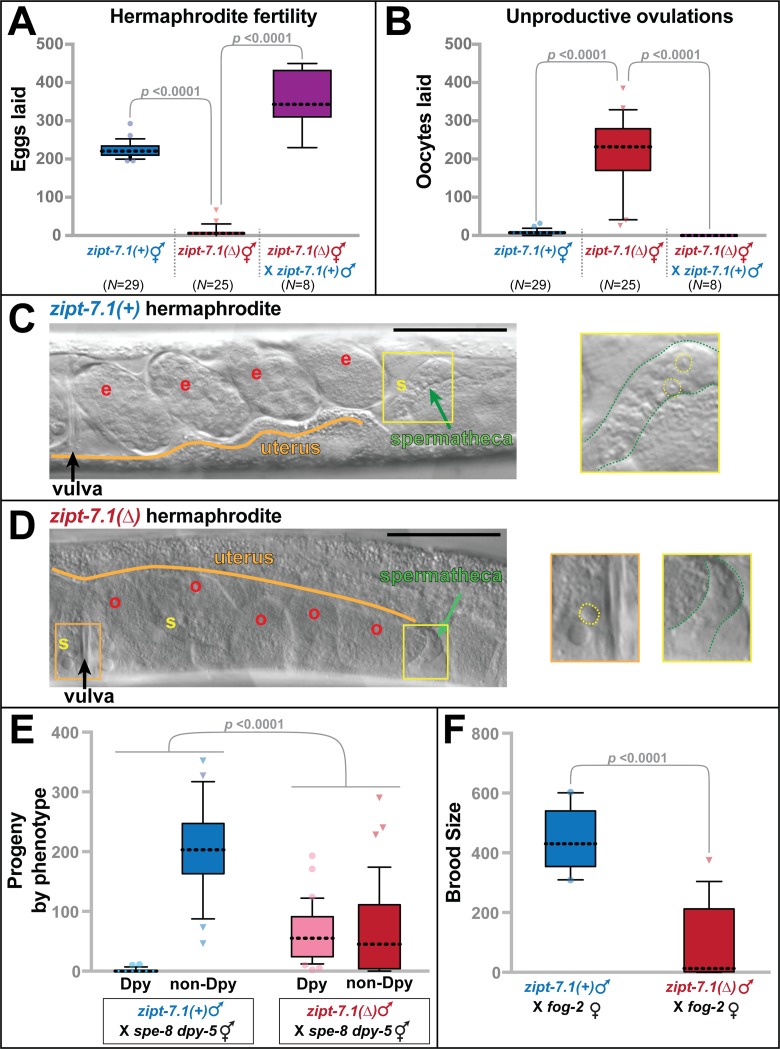
*zipt-7*.*1* is required for sperm activity in both sexes. (A,B) Values are total number of eggs (A) and unfertilized oocytes (B) laid by hermaphrodites in the same 5-day span. Box and whisker plots show the mean (dotted line), 25th to 75th percentiles (box), and 10th to 90th percentiles (whiskers). Points falling outside of this range are marked individually. *N* indicates number of broods scored. The deletion allele was *zipt-7*.*1(ok971)*, and all strains contained *him-5(e1490)*. (C) Photomicrograph of the uterus and spermatheca of a wild-type hermaphrodite that carried the *him-5(e1490)* mutation. A yellow “s” marks a group of sperm, and a red “e” marks each embryo. The inset shows a 4-fold expansion of the boxed region, which contains the spermatheca (green arrow in the main image; green outline in the inset). (D) Photomicrograph of a *zipt-7*.*1(ok971)* hermaphrodite that also carried the *him-5(e1490)* mutation. A yellow “s” marks each sperm in the uterus, and a red “o” marks each unfertilized oocyte. The area in the orange box is expanded 4-fold in the left inset, and the area in the yellow box is expanded 4-fold in the right inset to show the empty spermatheca. (C,D) In the insets, some sperm are indicated by dotted yellow circles. Anterior is left and ventral is down. Scale bars are 50 μm. (E) Total broods from crosses between either wild-type males and *spe-8 dpy-5* sterile hermaphrodites (*N* = 23) or *zipt-7*.*1(ok971)* males and *spe-8 dpy-5* sterile hermaphrodites (*N* = 39). All males carried the *him-5* mutation. (F) Total broods from crosses between either wild-type males and *fog-2* females (*N* = 13) or *zipt-7*.*1(ok971)* males and *fog-2* females (*N* = 14). All males carried the *him-5* mutation. In A, B, and F, statistical significance was calculated using the Mann-Whitney *U* test, whereas in E, it was determined with a 2 × 2 contingency table. The individual numerical values for panels A, B, E, and F can be found in S1 Data.

To analyze *zipt-7*.*1* function, we studied the null allele *ok971*, which deletes the entire coding region (Fig 1B). Whereas wild-type hermaphrodites had an average brood size of 225 self progeny, and individuals were invariably fertile, *zipt-7*.*1* mutants had significantly smaller broods, and most individuals were completely sterile (Fig 2A, S1A Fig). Thus, *zipt-7*.*1* loss-of-function causes a fully penetrant reduction in the number of self progeny and partially penetrant sterility. Furthermore, these mutant hermaphrodites laid large numbers of unfertilized oocytes (Fig 2B, S1A Fig), which implies that the MSP signal that stimulates ovulation is intact [13]. Because both of these defects were corrected by crossing *zipt-7*.*1(ok971)* hermaphrodites with wild-type males (Fig 2A and 2B), we infer that the mutant hermaphrodites make defective sperm but functional oocytes.

To characterize this fertility defect, we used differential interference contrast (DIC) optics to view live animals. In wild-type hermaphrodites, sperm actively moved into the two spermathecae. As a result, each ovulation resulted in fertilization and the release of a new embryo into the uterus (Fig 2C). By contrast, in *zipt-7*.*1* mutant hermaphrodites the spermathecae were empty and scattered spermatids and unfertilized oocytes were visible in the uterus (Fig 2D). We infer that the mutant sperm retained the ability to stimulate ovulation but were unable to migrate back to the spermathecae after being pushed into the uterus during ovulation [6].

To study male sperm, we used crosses with self-sterile hermaphrodites or females. We first tested the ability of male sperm to compete with *spe-8* hermaphrodite sperm, which fail to activate unless stimulated by male seminal fluid [14]. The wild-type male sperm competed efficiently with *Trans*-activated *spe-8* hermaphrodite sperm, fertilizing all of the oocytes and yielding only cross progeny. By contrast, the *zipt-7*.*1* male sperm competed poorly, fertilizing only a minority of the oocytes and resulting in numerous self progeny (Fig 2E).

To learn if the *zipt-7*.*1* male sperm were defective in competition or had an absolute decline in function, we measured the ability of *zipt-7*.*1* males to fertilize *fog-2* females, which make no self-sperm. Even though the *zipt-7*.*1* mutant sperm had no competition, we observed a dramatic decrease in successful fertilizations compared to wild-type males (Fig 2F). Thus, *zipt-7*.*1* activity promotes the function of sperm in both hermaphrodites and males, and it appears to regulate either activation or motility.

### *zipt-7*.*1* promotes sperm activation

**Fig 3:**
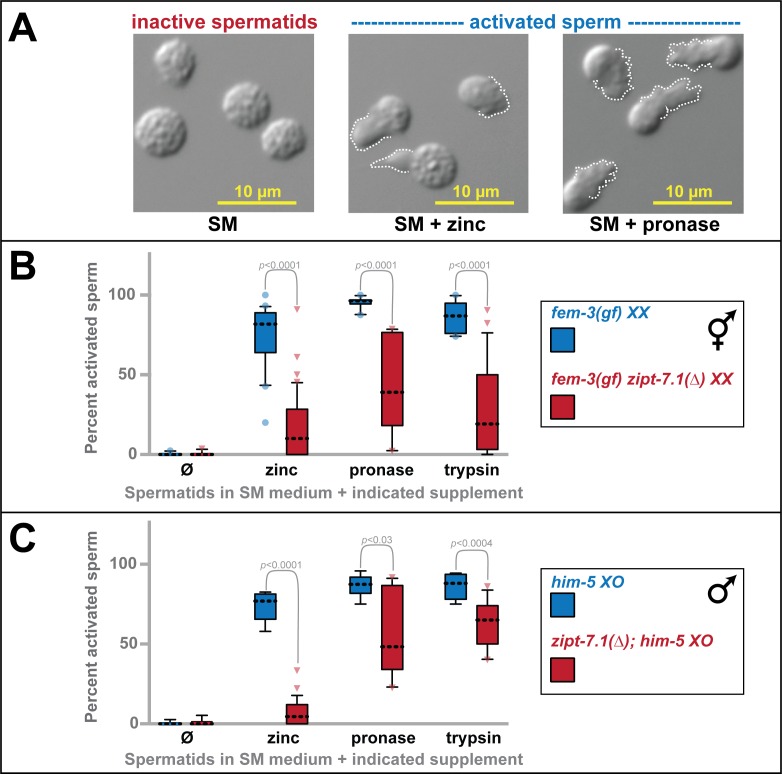
*zipt-7*.*1* controls sperm activation. (A) DIC photomicrographs of wild-type spermatids in standard SM medium (left) or SM medium supplemented with zinc (middle) or Pronase (right). Activation (or spermiogenesis) results in the extension of pseudopods, which are indicated with dotted white lines. (B,C) Using these morphological criteria, spermatids were scored in multiple small batches to determine which were active or inactive, with the average for each batch yielding one data point (*N* = 8–48 batches). Box and whisker conventions are described in Fig 2, and the Mann-Whitney *U* test was used for statistical comparisons. Alleles were *fem-3(q96*gf*)*, a mutation that causes hermaphrodites to produce sperm throughout their lives so as to increase sample sizes [15], *him-5(e1490)*, a mutation that increases the frequency of male progeny but has no other developmental effects [16], and *zipt-7*.*1(ok971)*. The individual numerical values for panels B and C can be found in S1 Data. DIC, differential interference contrast; SM, Sperm Medium.

Nematode spermatids remain round and immotile until they receive an activating signal, which causes them to extend a pseudopod and begin to crawl (Fig 3A) [4]. Sperm isolated by dissection from wild-type hermaphrodites displayed the extended pseudopods characteristic of in vivo activation. By contrast, those isolated from *zipt-7*.*1* hermaphrodites lacked pseudopods and appeared to be round, immotile spermatids, suggesting activation had not occurred (S1 Fig). Hence, we began studying the ability of *zipt-7*.*1* spermatids to activate.

The trypsin protease TRY-5 is an endogenous activating signal [8], and zinc [10], trypsin, and the protease mixture Pronase [9] can stimulate activation in vitro. To measure the response of spermatids to these signals, we dissected adult animals to release spermatids into sperm medium. Because wild-type hermaphrodites produce only small numbers of sperm prior to oogenesis, we analyzed *fem-3(q96)* mutants, which produce numerous sperm throughout their lives [15]. Spermatids dissected from *fem-3* hermaphrodites displayed robust activation in response to all three signals in vitro; by contrast, those dissected from *fem-3 zipt-7*.*1* double mutants displayed significantly lower levels of activation (Fig 3B). We repeated these experiments with spermatids isolated from males and obtained similar results (Fig 3C). Thus, *zipt-7*.*1* regulates activation.

Two points were notable. First, *zipt-7*.*1* mutant spermatids occasionally activated, indicating that this defect is not completely penetrant. This result might explain our observation that *zipt-7*.*1* sterility is also partially penetrant. Second, the least effective activator of *zipt-7*.*1* mutant spermatids was zinc, which is consistent with the model that *zipt-7*.*1* functions in zinc biology.

### ZIPT-7.1 is expressed in and functions in developing spermatocytes

**Fig 4:**
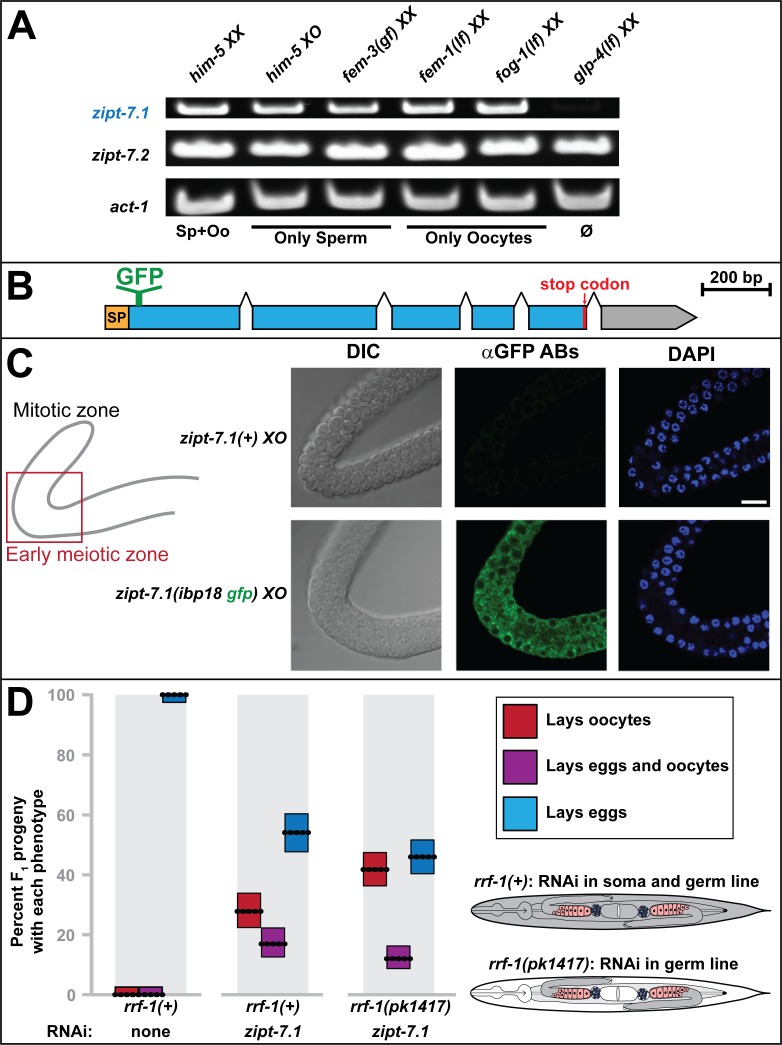
ZIPT-7.1 is expressed and functions in the germ line to control sperm activation. **(A)** Bands represent transcript levels determined by semiquantitative RT-PCR. Genotypes are labeled above, target transcripts at the left, and germ line phenotypes below; *act-1* is a loading control. For each lane, RNA was isolated from batches of five worms (see Materials and methods). The alleles were *him-5(e1490)*, *fem-3(q96)*, *fem-1(hc17*ts*)*, *fog-1(q253*ts*)*, and *glp-4(bn2*ts*)*. (B) Diagram illustrating the insertion site of the GFP coding sequence into the endogenous *zipt-7*.*1* locus to generate the *zipt-7*.*1(ibp18)* strain. (C) Sets of photomicrographs from two extruded male gonads. The location of the images in the gonad arm is indicated with a red box on the cartoon; distal is up and proximal to the right. The bright-field images show cell morphology (left), the antibody stain shows the expression of GFP::ZIPT-7.1 (middle), and the DAPI stain shows cell nuclei (right). Genotypes were *him-5(e1490)* and *him-5(e1490) zipt-7*.*1(ibp18)*. Scale bar is 10 μm. (D) Reproductive defects caused by *zipt-7*.*1* RNAi treatment. The F_1_ progeny of mothers injected with dsRNA were each scored for whether they laid eggs, oocytes, or both. Dotted lines show the average percentage for each phenotype and sum to 100%, the size of the boxes indicates 95% confidence limits, and the color indicates the phenotype according to the key. The *rrf-1* genotype and RNAi treatment are indicated at the bottom (*N* = 200, 248, and 315, left to right). Wild-type animals are susceptible to RNAi in germ cells and most somatic cells, whereas the *rrf-1* mutant animals are susceptible to RNAi in germ cells but resistant in most of the somatic cells [17–19], as indicated in the diagram. The individual numerical values for panel D can be found in S1 Data. DIC, differential interference contrast; dsRNA, double-stranded RNA; GFP, green fluorescent protein; Oo, oocyte; Sp, sperm; RNAi, RNA interference; RT-PCR, Reverse transcription polymerase chain reaction.

To determine the expression pattern of *zipt-7*.*1*, we used Reverse transcription polymerase chain reaction (RT-PCR) to analyze transcript levels in mutant strains that had altered germ cell fates. The *zipt-7*.*1* transcripts were readily detectable in animals containing only sperm or only oocytes, but almost undetectable in animals that lacked most germ cells (Fig 4A). These results suggest that *zipt-7*.*1* is predominantly expressed in the germ line or that its expression in other tissues depends on germ cells. By contrast, transcripts of the related gene *zipt-7*.*2* were readily detectable in animals that lacked most germ cells, indicating expression in somatic tissues.

It was technically challenging to visualize the ZIPT-7.1 protein in situ. Two different polyclonal antibodies raised against ZIPT-7.1 peptides were unable to detect ZIPT-7.1 expression in situ, although they could detect ZIPT-7.1 expressed in human cells (S3 Fig); thus, in vivo expression levels might be low. Multiple attempts to insert epitope tags into the endogenous locus were unsuccessful. Ultimately, we used gene editing to insert sequences encoding green fluorescent protein (GFP) into the endogenous *zipt-7*.*1* locus to encode a fusion protein with GFP inserted in the first predicted cytoplasmic loop, between amino acids 25 and 26 (Fig 4B). Animals homozygous for the modified allele developed normally, demonstrating that this GFP::ZIPT-7.1 fusion protein is functional. Protein expression could only be detected with anti-GFP antibodies, because the expression level was too low to observe GFP fluorescence. We found that GFP::ZIPT-7.1 expression levels were highest in developing spermatocytes, consistent with the analysis of transcript expression (Fig 4C). Furthermore, it appeared to be excluded from the nucleus and concentrated in puncta in the cytoplasm, suggesting that it localized to subcellular organelles. GFP::ZIPT-7.1 could not be visualized in spermatids or mature sperm with this protocol, which could be due to its low level of expression.

Based on these data, we hypothesized that *zipt-7*.*1* functions cell autonomously to promote sperm activation. To test this model, we used RNA interference (RNAi) in *rrf-1* mutants. Wild-type animals are susceptible to RNAi in the germ line and most somatic cells. By contrast, *rrf-1* function is required for RNAi to work in most of the somatic tissues, so *rrf-1(*−*)* mutants are mainly susceptible to RNAi in the germ line [17–19]. RNAi directed against *zipt-7*.*1* caused similar sterility in both wild-type and *rrf-1* mutant hermaphrodites (Fig 4D). Thus, *zipt-7*.*1* is necessary in the germ line to promote fertility.

Taking all of these results together, we conclude that ZIPT-7.1 functions in developing sperm to regulate activation, rather than in the soma to control an activating signal.

### ZIPT-7.1 regulates intracellular zinc levels

**Fig 5:**
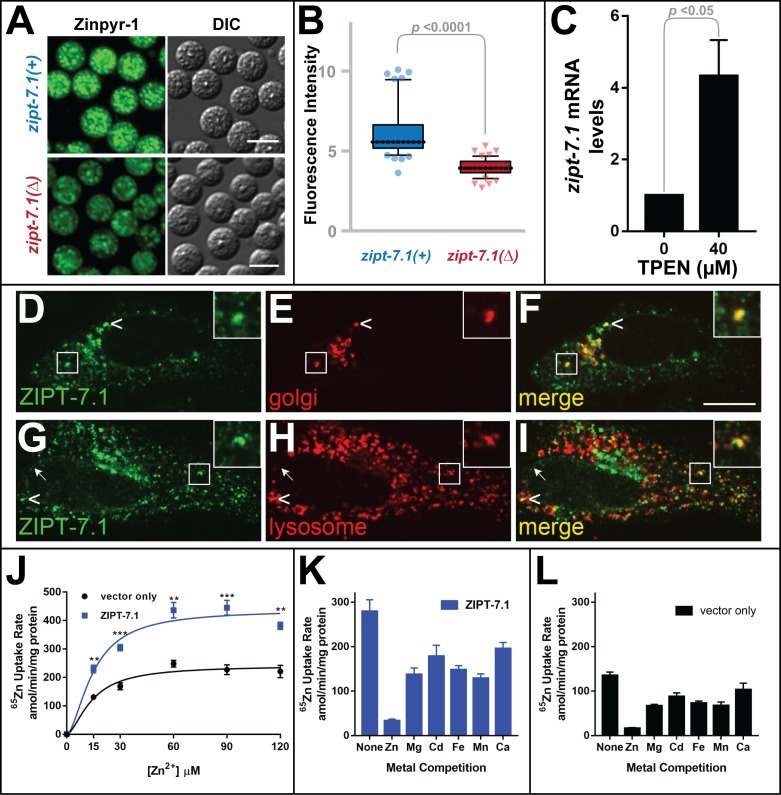
ZIPT-7.1 transports zinc in vitro and regulates zinc levels in vivo. (A) Photomicrographs of spermatids isolated from *him-5* males (upper) or *him-5 zipt-7*.*1(ok971)* males (lower). Spermatids were stained with Zinpyr-1 and visualized with fluorescence to reveal labile zinc (left) and DIC optics to show morphology (right). Scale bar is 5 μm. (B) Fluorescence intensity is (F-F_0_)/F_0_, where F is the fluorescence level of each spermatid (*N* = 50), and F_0_ is background. Box and whiskers conventions are described in Fig 2; comparisons used the Mann-Whitney *U* test. (C) Wild-type animals were cultured with 0 or 40 μM TPEN, a zinc chelator. RNA from a mixed-stage population was analyzed by qRT-PCR. Mean mRNA levels are shown relative to those for 0 μM TPEN, which was set to 1; error bars indicate SE (*N* = 4 trials). (D–I) Human HeLa cells expressing ZIPT-7.1 were double labeled with antibodies against ZIPT-7.1 (D, G, green) and the *Cis*-Golgi marker GM130 (E, red) or the lysosomal marker LMP2 (H, red). Confocal fluorescence micrographs of representative cells show ZIPT-7.1 localization, which appears to be at the nuclear envelope, Golgi, and lysosomes. Arrowheads indicate apparent colocalization of ZIPT-7.1 with GM130 or LMP2 (yellow), and the arrow indicates nuclear envelope localization. The boxed area highlights apparent colocalization and is enlarged 2.3-fold in the inset. Scale bar is 5 μm. (J–L) Human HEK293T cells expressing ZIPT-7.1 or an empty vector control were incubated with radioactive zinc solution, which contains a trace amount of radioactive ^65^Zn, and the rate of zinc uptake was determined. Values are mean and SE (*N* = 4 trials). (J) Uptake as a function of total zinc concentration in the medium. ZIPT-7.1 expression significantly increased uptake (** *p* < 0.01; *** *p* < 0.001). (K, L) Competition experiments with nonradioactive ions were used to establish metal specificity. Assays contained 30 μM radioactive zinc solution and 300 μM nonradioactive zinc, magnesium, cadmium, iron, manganese, or calcium. For cells expressing ZIPT-7.1, comparison of the different metals reveals that zinc was significantly more effective as a competitor than other ions (Fe, Ca, *p* < 0.001; Mg, Cd, Mn, *p* < 0.01). For control cells, which reveal uptake mediated by endogenous proteins, comparison of the different metals reveals that zinc was also significantly more effective as a competitor than other ions (Mg, Fe, *p* < 0.001; Cd, Mn, Ca, *p* < 0.01). The individual numerical values for panels B, C, J, K, and L can be found in S1 Data. DIC, differential interference contrast; qRT-PCR, quantitative RT-PCR; TPEN, N,N,N',N'-tetrakis(2-pyridinylmethyl)-1,2-ethanediamine.

Many ZIP family proteins specifically transport zinc, but some transport iron or other metals. Thus, we investigated the role of ZIPT-7.1 in zinc biology. To analyze levels of labile zinc in vivo, we isolated male spermatids and stained them with Zinpyr-1, a dye that fluoresces when it binds zinc [20]. Wild-type spermatids displayed punctate fluorescence (Fig 5A) [10]. Some puncta colocalized with the dye LysoTracker, suggesting they were membranous organelles, and others colocalized with the dye MitoTracker, suggesting they were mitochondria (S2 Fig). Although *zipt-7*.*1* mutant spermatids displayed a similar pattern of fluorescence, the intensity of the fluorescence was significantly lower (Fig 5A and 5B). Because this difference is detectable after spermatogenesis is complete but before activation has occurred, we infer that *zipt-7*.*1* promotes the accumulation of intracellular zinc during spermatogenesis. Following activation, the mature sperm extend a pseudopod that does not display labile zinc, membranous organelles, nor mitochondria (S2 Fig).

If *zipt-7*.*1* functions in zinc biology, then its expression might be regulated by the level of available zinc. To test this prediction, we cultured wild-type animals with the zinc-specific chelator N,N,N',N'-tetrakis(2-pyridinylmethyl)-1,2-ethanediamine (TPEN) to induce zinc deficiency and analyzed *zipt-7*.*1* transcript levels. Although the expression of control genes did not change, levels of *zipt-7*.*1* mRNA increased 4-fold (Fig 5C), consistent with the model that *zipt-7*.*1* plays an in vivo role in zinc biology. This finding is consistent with a recent report by Dietrich and colleagues [21] that the *zipt-7*.*1* locus contains a low zinc activation (LZA) enhancer motif that mediates transcriptional activation in response to low dietary zinc.

To test the prediction that ZIPT-7.1 transports zinc, we expressed it in mammalian cells and measured its subcellular localization and zinc uptake using radioactive ^65^Zn. We used human HeLa cells for localization studies because of their well-defined cellular architecture. Antibody staining revealed that ZIPT-7.1 was expressed in a punctate pattern, and some puncta appeared to colocalize with the *Cis*-Golgi marker GM130 and the lysosomal marker LMP2. In addition, some ZIPT-7.1 appeared to be localized on the nuclear envelope (Fig 5D–5I, S3 Fig). We used human HEK293T cells for uptake studies because of their high efficiency of transfection. The expression of ZIPT-7.1 significantly increased the uptake of radioactive ^65^Zn from the medium (Fig 5J–5L). Furthermore, this transport activity was zinc specific, because it was effectively competed by nonradioactive zinc but not by several other metal ions (Fig 5K and 5L).

Thus, three lines of evidence suggest that ZIPT-7.1 is a zinc-specific transporter. (1) The expression of *zipt-7*.*1* transcripts in nematodes is regulated by zinc, (2) it controls intracellular zinc levels in developing spermatids, and (3) it is capable of specifically transporting zinc across membranes when assayed in mammalian cells.

### The control of sperm activation by ZIPT-7.1 is conserved in other nematodes

**Fig 6:**
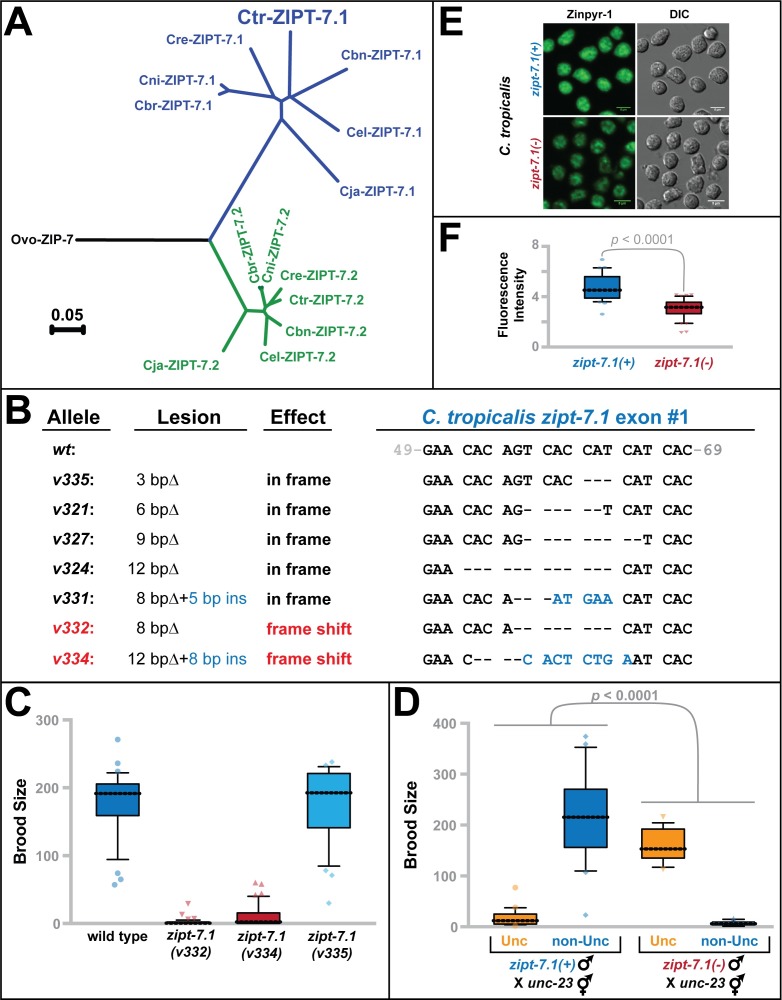
Regulation of sperm activation by ZIPT-7.1 is conserved in nematodes. (A) Maximum likelihood phylogeny of all ZIP7 homologs identified in eight nematode species: *Caenorhabditis briggsae* (Cbr), *C*. *nigoni* (Cni), *C*. *remanei* (Cre), *C*. *tropicalis* (Ctr), *C*. *brenneri* (Cbn), *C*. *elegans* (Cel), *C*. *japonica* (Cja), and *Onchocerca volvulus* (Ovo). Sequences were obtained from wormbase.org and aligned using ClustalX, and calculations were done using PhyML with 100 bootstrap replications. Blue indicates the ZIPT-7.1 subfamily and green the ZIPT-7.2 subfamily. (B) *C*. *tropicalis zipt-7*.*1* mutations produced by gene editing. Gray numbers indicate positions in the coding sequence of the gene. Deleted nucleotides are shown as dashes, and inserted nucleotides are blue. We selected the frameshift alleles *v332* and *v334* as representative null alleles and the in-frame deletion *v335* as a nearly wild-type control. (C, D) Brood sizes of *ctr-zipt-7*.*1* hermaphrodites and males, presented as in Fig 2. JU1373 is the wild-type strain of *C*. *tropicalis*. For D–F, wild-type males were *him-8(v287)* and mutant males were *him-8(v287); zipt-7*.*1(v332)*. (E) Photomicrographs of spermatids stained with Zinpyr-1 to reveal labile zinc levels, as in Fig 5A. Scale bars are 5 μm. (F) Quantitation of fluorescence intensity, as in Fig 5B. The individual numerical values for panels C, D, and F can be found in S1 Data. Cbn, *C*. *brenneri*; Cbr, *C*. *briggsae*; Cel, *C*. *elegans*; Cja, *C*. *japonica*; Cni, *C*. *nigoni*; Cre, *C*. *remanei*; Ctr, *C*. *tropicalis*; Ovo, *Onchocerca volvulus*.

*C*. *elegans* ZIPT-7.1 and ZIPT-7.2 are both closely related to a single human protein, ZIP7, indicating that a primordial gene duplicated and diverged in the nematode lineage. To investigate this gene duplication, we analyzed the genomes of related nematodes. Orthologs of both genes are present throughout the *elegans* group of the genus *Caenorhabditis*, but the nematode *Onchocerca volvulus* contained only a single ZIP7 homolog (Fig 6A) [22]. We conclude that the duplication and functional divergence of ZIPT-7.1 and ZIPT-7.2 occurred relatively recently during nematode evolution.

To see if the function of *zipt-7*.*1* is conserved, we used gene editing to alter exon #1 of the *zipt-7*.*1* gene in the related hermaphroditic species *C*. *tropicalis* [23–25]. We recovered seven mutant alleles, including two frameshift mutations predicted to eliminate gene function (Fig 6B). Both of these frameshift mutations caused hermaphrodite sterility (Fig 6C). We tested one allele in males, for whom it also caused sterility (Fig 6D). Finally, Zinpyr-1 staining showed that this mutation resulted in reduced zinc accumulation in spermatids (Fig 6E and 6F). Thus, all three *zipt-7*.*1(*−*)* phenotypes observed in *C*. *tropicalis* are similar to those already described for *C*. *elegans*. These results suggest that *zipt-7*.*1* has a conserved function in *Caenorhabditis*—it promotes sperm activation by regulating zinc.

### *zipt-7*.*1* acts downstream of *spe-6*, and ZIPT-7.1 binds to SPE-4

In *C*. *elegans*, multiple genes can be mutated to block sperm activation or cause constitutive activation [4]. These genes have been organized into two genetic pathways, and the subcellular localizations of several proteins have been identified. In hermaphrodites, sperm activation is controlled by an unknown signal that acts through five proteins located at the cell membrane of the spermatid [4]. Mutations in any of these five genes (*spe-8*, *spe-12*, *spe-19*, *spe-27*, or *spe-29*) prevent activation. By contrast, mutations in *spe-6* and *spe-4* suppress the defective activation phenotype produced by these *spe-8* group genes and also cause male sperm to activate prematurely, prior to ejaculation [26, 27]. These results suggest that *spe-4* and *spe-6* act downstream of *spe-8* and its partners, and *spe-4* and *spe-6* currently define the downstream end points of this sperm activation pathway.

**Fig 7:**
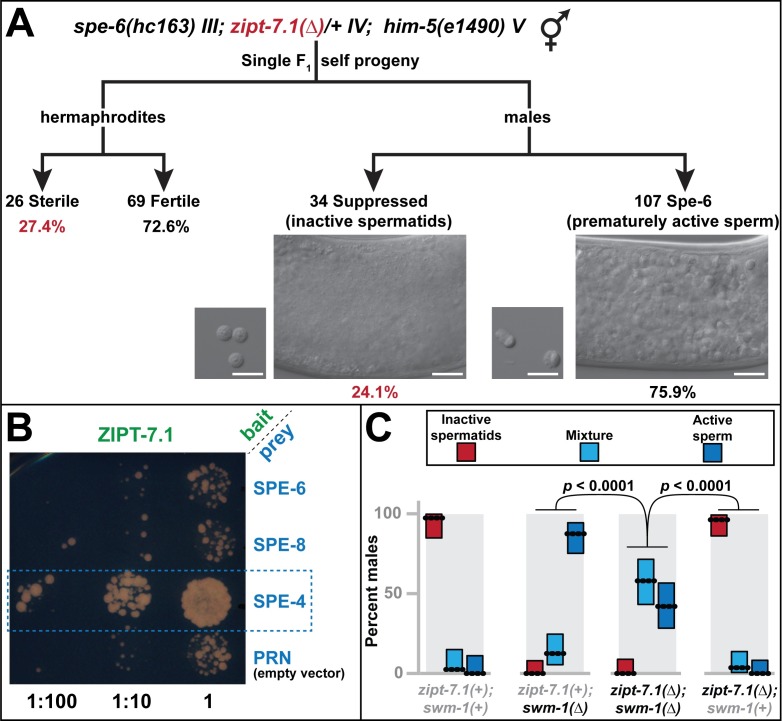
Genetic and physical interactions of ZIPT-7.1 with the SPE-8 activation pathway. (A) Self progeny of *spe-6(hc163)* III*; zipt-7*.*1(ok971)/+* IV*; him-5(e1490)* V hermaphrodites were analyzed for active sperm in the male gonad (the *spe-6(*−*)* phenotype) or hermaphrodite sterility (the *zipt-7*.*1(*−*)* phenotype). About 25% of the animals were predicted to be homozygous *zipt-7*.*1(*−*)* mutants (red values). The number and fraction of progeny of each sex displaying these phenotypes are shown. Photomicrographs show DIC images of representative male gonads (large panel) and dissected sperm (small panel). Scale bars are 10 μm. About 25% of hermaphrodites were sterile, indicating that *spe-6(*−*)* did not suppress the *zipt-7*.*1(*−*)* phenotype. About 25% of males did not have prematurely active sperm, indicating that *zipt-7*.*1(*−*)* suppressed the *spe-6(*−*)* phenotype. (B) Photomicrograph showing the density of yeast colonies at three plating dilutions. The split-ubiquitin yeast two-hybrid study was conducted with ZIPT-7.1 expressed from the bait plasmid (green) and other proteins expressed from the prey plasmid (blue). The box marks robust colony growth with SPE-4, indicating a protein–protein interaction. (C) Males were classified as having inactive spermatids (red), characteristic of both *zipt-7*.*1(*−*)* and the wild type, active sperm (dark blue), characteristic of *swm-1(*−*)*, or a mixture of spermatids and active sperm (light blue). From left to right, *N* = 39, 56, 50, and 54; dotted lines show the percentage of animals that displayed each phenotype, and boxes show 95% confidence intervals. All strains included *him-5(e1490)* and either *zipt-7*.*1(ok971)* or *swm-1(ok1193)*, as indicated. The individual numerical values for panels A and C can be found in S1 Data. DIC, differential interference contrast.

**Fig 8:**
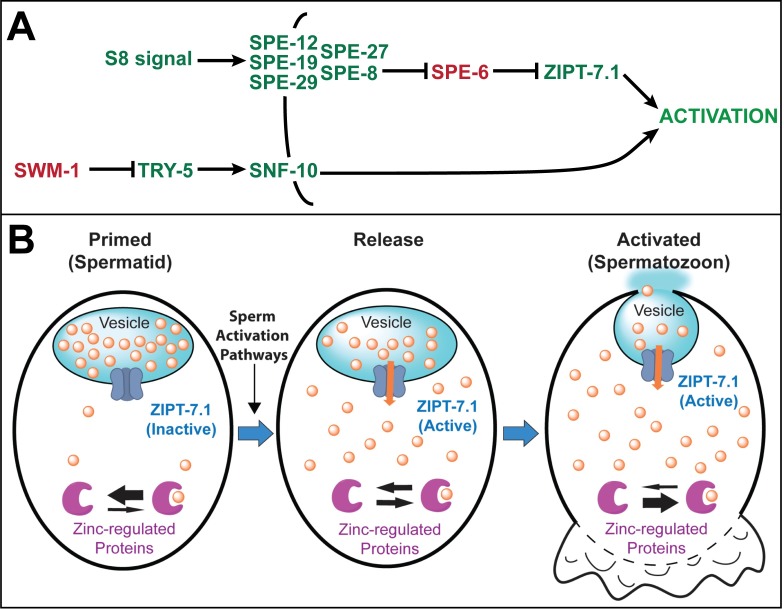
Model for ZIPT-7.1 function. (A) Model of the genetic function of *zipt-7*.*1*. The black line shows the plasma membrane, and arrows and bars indicate positive and negative interactions, respectively. Genes that mutate to sperm activation defective or constitutive are green or red, respectively. (B) Model of the biochemical function of ZIPT-7.1 at three times during sperm activation: (1) Primed spermatid: prior to activation, spermatids are primed to respond—ZIPT-7.1 (dark blue) is inactive and localized to the membrane of a vesicle that has a high internal concentration of stored zinc (orange circles) and is presumably the membranous organelle (light blue). The cytoplasm has a low concentration of zinc, and the equilibrium of zinc-regulated proteins (purple) is shifted towards unbound. (2) Release: a sperm-activating signal results in activation of ZIPT-7.1 (orange arrow), zinc begins to flow into the cytoplasm, and zinc-regulated proteins begin binding zinc. (3) Activated spermatozoon: the cytoplasmic concentration of zinc is now high, and zinc-regulated proteins are bound to zinc. The pseudopod extension begins (lower), and the membranous organelles fuse with the plasma membrane. Fusion of membranous organelles is based on previously published observations of this event [28], and our results do not directly address whether ZIPT-7.1 is relocalized during this process.

To position *zipt-7*.*1* in this pathway, we generated double mutants with *spe-4(hc196)* and *spe-6(hc163)*. Germ cells in the *spe-4; zipt-7*.*1* double mutant arrested as abnormal primary spermatocytes that failed to divide. Because they made no sperm that could be tested for activation, this approach was not informative. By contrast, *spe-6* mutant males displayed prematurely active sperm in their spermathecae [26], but *spe-6(*−*); zipt-7*.*1(*−*)* males did not (Fig 7A). Furthermore, *spe-6(*−*); zipt-7*.*1(*−*)* hermaphrodites were self-sterile, suggesting that *zipt-7*.*1* functions downstream of *spe-6* in both sexes, or that these two genes act in parallel (Fig 7A). By contrast, *spe-8(*−*)*; *spe-6(*−*)* hermaphrodites are self-fertile [26]. These results distinguish *zipt-7*.*1* from the *spe-8* group and suggest that *zipt-7*.*1* functions downstream of *spe-6*, at the end of the sperm activation pathway (Fig 8A).

In males, sperm can also be activated by the extracellular protease TRY-5, which is likely to act through the membrane protein SNF-10 [8, 29]. Prior to ejaculation, TRY-5 is inhibited by the SWM-1 protease inhibitor, which prevents premature activation [30]. Thus, *swm-1* mutant males have abnormally active sperm crawling inside the reproductive tract, similar to *spe-6* or *spe-4* mutant males. The phenotype of *zipt-7*.*1(*−*); swm-1(*−*)* double mutant males was intermediate between that of each single mutant (Fig 7C), so *zipt-7*.*1* might function in parallel to the *try-5* pathway (Fig 8A).

To complement these genetic experiments, we performed biochemical studies using the split-ubiquitin two-hybrid system (Fig 7B, S4 Fig). ZIPT-7.1 interacted robustly with SPE-4, a presenilin localized to the membrane of the membranous organelles [27], but not with SPE-6, SPE-8, SPE-19, SPE-27, or SPE-43. Thus, SPE-4 might directly inhibit ZIPT-7.1 function in spermatids to prevent premature sperm activation, and relief of this inhibition by the sperm activation pathway might allow ZIPT-7.1 to transport zinc, elevating the zinc concentration in the cytoplasm and promoting sperm activation.

## Discussion

### *zipt-7*.*1* encodes a ZIP family zinc transporter that plays a critical role in sperm activation

The analysis of three mutations demonstrates that *zipt-7*.*1* promotes sperm activation. Two are molecular null alleles—*hc130* eliminates the start codon and *ok971* deletes the entire coding region—whereas *as42* changes a glycine to glutamic acid in a predicted transmembrane domain. All three mutations severely reduced production of hermaphrodite self progeny, and rescue by crossing with wild-type males indicates a defect in hermaphrodite sperm. There is also a defect in male sperm, because *zipt-7*.*1* mutant males were impaired in fertilizing hermaphrodites. Spermatids dissected from *zipt-7*.*1* hermaphrodites had failed to activate, and mutant spermatids from either sex responded poorly to triggering agents like zinc, Pronase, or trypsin, pinpointing the problem to sperm activation.

Whereas other animals such as fruit flies and humans have a single ZIP7 gene, *C*. *elegans* has two—*zipt-7*.*1* and *zipt-7*.*2*—suggesting a gene duplication occurred in nematodes. These two genes have separate functions, because the *zipt-7*.*2(ok960)* mutation causes lethality (wormbase.org), indicating that somatic functions of ZIP7 are necessary for survival and are provided by *zipt-7*.*2*. To determine if these specializations have been conserved in *Caenorhabditis*, we obtained mutations of *zipt-7*.*1* in the related nematode *C*. *tropicalis*. As observed with *C*. *elegans*, the *C*. *tropicalis zipt-7*.*1* mutations caused a decrease in hermaphrodite and male fertility, indicating a sperm defect in both sexes, and abnormal zinc staining of spermatids, indicating a defect in zinc biology. Thus, the duplication of the ZIP7 genes during *Caenorhabditis* evolution appears to have been accompanied by the specialization of *zipt-7*.*1* to control sperm development. As a result, *C*. *elegans* z*ipt-7*.*1* is a powerful model for studying ZIP7 genes during reproduction, because the essential functions of ZIP7 transporters are covered by its sister, *zipt-7*.*2*, thereby avoiding the complexities of pleiotropic phenotypes and inviable mutants.

*zipt-7*.*1* is the first zinc transporter to be implicated in sperm activation, and this discovery opens up exciting new models for the role of zinc in this process of rapid cell differentiation. We hypothesize that ZIPT-7.1 mediates the regulated release of zinc from the lumen of intracellular vesicles, and that released zinc functions as a second messenger to promote sperm activation (Fig 8B). A variety of approaches have led to the identification of multiple genes that are involved in sperm development in worms, but *zipt-7*.*1* is the first of these genes to have a clear role in zinc biology. In other animals, such as *Drosophila* and vertebrates, zinc transporters have not been demonstrated to function in sperm activation, highlighting the novelty of this discovery.

### Zinc may act as a second messenger to induce sperm activation in nematodes

Sperm activation is a unique cell differentiation process that is rapidly induced in response to extracellular cues but does not involve transcriptional changes. Despite extensive investigations in multiple species, the signaling cascade that initiates sperm activation remains poorly defined. Here we propose that ZIPT-7.1 mediates the release of zinc from intracellular organelles in spermatids and that zinc may function as a second messenger that promotes sperm activation (Fig 8B).

*C*. *elegans* ZIPT-7.1 is similar to human ZIP7 and *Drosophila* Catsup, which implies that it transports a divalent cation. When expressed in mammalian cells, ZIPT-7.1 robustly increased zinc uptake, and competition experiments demonstrated that this transport was relatively specific for zinc. Thus, the zinc transport activity of ZIPT-7.1 was predicted by homology and confirmed by biochemical studies. Furthermore, ZIPT-7.1 protein localized primarily to internal membranes of mammalian cells, including the lysosome and Golgi, so it is likely to control release of zinc from internal stores. This localization pattern is similar to that of vertebrate ZIP7 [31], which implies that it is an evolutionarily conserved property of ZIP7 family members.

In nematodes, the expression and function of *zipt-7*.*1* are restricted to germ cells. First, *zipt-7*.*1* mRNA was predominantly expressed in the germ line. Second, a GFP::ZIPT-1 fusion protein expressed from the endogenous locus was localized in developing spermatocytes. Third, *zipt-7*.*1(RNAi)* caused sterility in an *rrf-1* mutant background, in which sensitivity is restricted primarily to the germ line [17–19], indicating that *zipt-7*.*1* functions in germ cells. These results suggest that *zipt-7*.*1* does not act in the soma to produce an activation signal but in sperm to mediate their response.

Two additional observations link *zipt-7*.*1* to zinc biology. First, *zipt-7*.*1* mRNA levels increased in response to zinc deficiency. Dietrich and colleagues [21] discovered an LZA enhancer element in the *zipt-7*.*1* gene that likely mediates this regulatory response. Second, the levels of labile zinc were reduced in spermatids from mutant animals, showing that *zipt-7*.*1* is required for the uptake and storage of wild-type levels of zinc in developing spermatocytes. Taken together, these observations pinpoint the expression pattern of ZIPT-7.1 (gonad), its site of action (gonad), its biochemical activity (zinc transporter increasing cytoplasmic zinc), and its subcellular localization (primarily internal membranes).

### ZIPT-7.1 interacts with the presenilin SPE-4

Extensive genetic and molecular studies have identified two pathways required for nematode sperm activation, referred to as the SPE-8 and TRY-5 pathways [4]. Both pathways contain negative regulatory genes that, when mutated, cause spermatids to activate constitutively and prematurely: *spe-6* and *spe-4* in the SPE-8 pathway and *swm-1* in the TRY-5 pathway. We took advantage of these alleles to perform genetic epistasis studies. A *zipt-7*.*1* mutation strongly suppressed the *spe-6* phenotype, suggesting that *zipt-7*.*1* acts downstream of *spe-6* if these genes act in a linear pathway. It remains possible that they function in parallel.

To characterize ZIPT-7.1 interactions with the SPE-8 pathway further, we used the yeast two-hybrid system to investigate protein–protein interactions. ZIPT-7.1 specifically bound SPE-4, a nematode presenilin that localizes to internal membranes in spermatids [32]. The role of presenilins in the regulation of zinc has formed an important area of research in Alzheimer disease for many years [33]. The fact that SPE-4 also acts late in the sperm activation process [27] and can bind ZIPT-7.1 supports the model that ZIPT-7.1 functions downstream of other known proteins in the SPE-8 pathway. Taken together, these results are consistent with the model that ZIPT-7.1 acts inside spermatids at the end of the known SPE-8 pathway to transmit an extracellular signal that triggers sperm activation (Fig 8A).

### Zinc signaling might play a conserved role in sperm activation

The effects of zinc on sperm have been investigated in several animals, including vertebrates (human, mouse, hamster), sea urchin, and *C*. *elegans*. Our results extend previous studies by (1) identifying the relevant zinc transporter and (2) providing a unified model for the function of zinc during sperm activation.

Sea urchin spermatids are normally ejaculated into sea water, and diluting them into sea water triggers activation and sperm motility [34]. Further studies demonstrated that zinc is the active ingredient in sea water and that zinc causes an increase in intracellular pH and intracellular calcium levels, triggering the acrosome reaction, a membrane fusion event [34, 35]. However, the mechanism by which zinc activates sea urchin sperm has not been defined, and no zinc transporters have thus far been implicated. The role of zinc in vertebrate sperm activation has been controversial; experiments with zinc chelators and the addition of supplemental zinc have suggested a variety of roles for zinc, indicating its importance, but specific functions have remained elusive [36].

High levels of extracellular zinc can activate *C*. *elegans* sperm in vitro [10], but the direct mechanism was not previously defined. We propose that physiological sperm activation involves zinc release from intracellular stores, which increases the cytoplasmic concentration of zinc and causes activation. High extracellular zinc leads to zinc entry into spermatids, thereby mimicking this physiological signal. The *C*. *elegans* model is similar to sea urchins in that rising concentrations of zinc stimulate sperm activation, with the difference that extracellular zinc is the physiological source in sea urchins, whereas intracellular stores are likely to be the physiological source in *C*. *elegans*. Although high levels of extracellular zinc can activate *C*. *elegans* sperm in a *zipt-7*.*1*–dependent manner in vitro, our results suggest this is unlikely to be the physiological trigger, because *zipt-7*.*1* acts downstream of SPE-8 pathway proteins located at the spermatid membrane.

### Sperm activation opens up new biology for zinc signaling

Well-established functions for zinc involve stable binding to proteins to influence tertiary structure or facilitate catalysis. In addition, zinc has been proposed to act as a second messenger, like calcium [37], but this function is just beginning to be explored and many questions remain. Proposed examples of zinc signaling can be divided into extracellular and intracellular. In the vertebrate nervous system, zinc is concentrated in synaptic vesicles with neurotransmitters and released into the synaptic cleft upon nerve stimulation, where it may modulate the activity of neurotransmitter receptors [11]. The second example of extracellular release is the “zinc spark” that has been visualized during mammalian oocyte fertilization [38]. This spark is caused by the synchronous fusion of many zinc-containing vesicles. In both cases, zinc is released to the extracellular space by vesicle fusion. By contrast, Yamasaki and colleagues visualized an intracellular “zinc wave” in vertebrate mast cells that had been stimulated to undergo degranulation by an extracellular ligand [39]. This zinc wave appeared to originate from the endoplasmic reticulum. In addition, a rapid increase in cytoplasmic zinc has been observed in T lymphocytes and leukocytes responding to extracellular signals [40, 41]. Finally, Hogstrand and colleagues proposed that the vertebrate ZIP7 is localized to intracellular membranes and mediates a zinc signal in breast epithelial cells [42].

The results presented here identify a new biological system for zinc signaling—sperm activation. It is intriguing that the “zinc wave” in mast cells mediates degranulation, a vesicle fusion event, and the zinc signal in sperm also mediates vesicle fusion. These results raise the possibility that intracellular zinc signals have a conserved function in promoting vesicle fusion. Our results are consistent with the model of vertebrate ZIP7 mediating an intracellular zinc signal, although the cell type and biological consequences are entirely different. Because nematode sperm activation can be dissected by forward and reverse genetics and manipulated both in vivo and in vitro, it offers a powerful model to define mechanisms of zinc signaling.

### Multiple ion fluxes during sperm activation

A critical question in sperm biology is how the rapid and dramatic changes that define activation are controlled without transcriptional regulation. The answer may involve converging lines of research on ion fluxes. First, it is well established for many animals, including nematodes, that intracellular pH increases during sperm activation [9]. Lishko and Kirichok identified a voltage-gated proton channel that mediates this pH rise in human sperm [43]. Second, calcium signaling has been repeatedly implicated in sperm activation and plays an important role in nematodes [44, 45]. The CatSper channel mediates this increase in intracellular calcium in mammalian sperm [46]. *C*. *elegans* does not have an obvious homolog of CatSper, but they do contain multiple predicted calcium channels; thus, calcium signaling in worms could be mediated by an alternative channel [47, 48]. Third, we propose here that zinc is a second messenger that activates sperm, and ZIPT-7.1 is the relevant transporter.

Considering our results in light of the documented roles of other ions, we speculate that sperm activation might be caused by a coordinated change in the cytoplasmic levels of three different ions: H^+^, Ca^++^, and Zn^++^. According to this model, a sperm activation signal results in the activation of an H^+^ exporter, a calcium importer, and the zinc importer ZIPT-7.1. The activity of these transporters changes the cytoplasm from a high concentration of H^+^ and low concentrations of Ca^++^ and Zn^++^ (the spermatid state) to a low concentration of H^+^ and high concentrations of Ca^++^ and Zn^++^ (the activated state). Changes in protein activity could result from alterations of pH and interactions with zinc and calcium cofactors without the need for altered transcription. Individual proteins might be influenced by one or more of these ion changes, and the combination of all three ion changes might rapidly alter the activity of a large number of proteins. Future work will be required to identify specific target proteins and determine whether pH, calcium, zinc, or a combination causes changes of protein activity.

## Materials and methods

### Worm strains

*C*. *elegans* strains were derived from Bristol N2 [49]. They include *fog-1(q253)* I, *glp-4(bn2)* I, *rrf-1(pk1417)* I, *Dsp2/spe-8(hc53) dpy-5* I, *spe-6(hc163) dpy-18(e364)* III, *dpy-13(e184)* IV, *zipt-7*.*1(hc130)* IV [informally referred to as *spe-24(hc130)*], *zipt-7*.*1(ok971)* IV [informally referred to as *hke-4*.*1(ok971)*], *zipt-7*.*1(as42)* IV, *fem-3(q96)* IV, *fem-1(hc17)* IV, *swm-1(ok1193)* V, *him-5(e1490)* V, and *fog-2 (q71)* V. Double or triple mutants made in this study include (1) *zipt-7*.*1(ok971)* IV; *him-5(e1490)* V, (2) *swm-1(ok1193) him-5(e1490)* V, (3) *zipt-7*.*1(ok971) fem-3(q96)* IV, (4) *zipt-7*.*1(ok971)* IV; *swm-1(ok1193) him-5(e1490)* V, and (5) *spe-6(hc163) dpy-18(e364)* III; *zipt-7*.*1(ok971)* IV. The *zipt-7*.*1(ibp18 gfp insertion)* strain was produced using CRISPR genome editing technology [50].

*C*. *tropicalis* mutants were derived from the wild isolate JU1373 and include *him-8(v287)*, *unc-23(v277)*, and *try-5(v275)* [25, 51]. The *zipt-7*.*1* mutations *v332*, *v334*, and *v335* described here were made using TALENs [25].

### Identification of *zipt-7*.*1(hc130)* by whole genome sequencing and hybrid analysis

Dpy hermaphrodites from the strain *hc130 dpy-13(e184)*/nT1 were crossed with N2 males to separate the *hc130* allele from *dpy-13*. Next, sterile, non-Dpy hermaphrodites isolated from the F_2_ were crossed with males from the polymorphic strain CB4856 [52, 53]. Finally, 480 F_2_ hermaphrodites from this cross were picked at the L4 stage to individual wells of 24-well plates. Each worm was scored for sterility the following day, and about 80 sterile F_2_ hermaphrodites were combined for isolation of sheared genomic DNA, as describe by Smith and colleagues [54].

We used 10 ng of fragmented DNA to prepare a library using the Hyper Prep Kit (KAPA Biosystems), as instructed by the manufacturer. Next, fragments sized 300–500 bp were selected using the QIAQuick Gel Extraction Kit (Qiagen) and sequenced on an Illumina HiSeq2000 instrument using the 50-cycles, single-end mode. We obtained 8,598,343 reads.

Data were analyzed at usegalaxy.org using the CloudMap Hawaiian Variant Mapping with WGS Data tool [55, 56]. The top candidate for the *hc130* mutation was a G to A transition in the start ATG codon of *zipt-7*.*1*. Sanger DNA sequencing confirmed the presence of this mutation in an *hc130 dpy-13(e184)* strain but not in our N2 strain.

### Protein alignment analysis

The following protein sequences are aligned in Fig 1D, identified by species, isoform, and NCBI reference sequence code: CeZIPT-7.1 (*C*. *elegans*, NP_503070.2); CeZIPT-7.2 (*C*. *elegans*, isoform 2, NP_510563.2); DmCATSUP (*D*. *melanogaster*, NP_524931.1); and HsZIP7 (*H*. *sapiens*, isoform 1 precursor, NP_008910.2). The alignment was performed with ClustalX.

### Fertility assays

To measure hermaphrodite fertility, we placed individual L4 animals on freshly seeded dishes, transferred them to new dishes every 8–16 hours for 5 days, and scored the number of fertilized eggs and unfertilized oocytes on each dish. To measure *C*. *elegans* male fertility, we used two assays. First, individual males were crossed to individual *spe-8(hc53); dpy-5* L4 hermaphrodites for 24 hours. The male was removed, the hermaphrodite was transferred to a new dish every 24 hours, and the offspring were scored as Dpy (self) or non-Dpy (cross) progeny upon reaching adulthood. Second, males were crossed to *fog-2(q71)* L4 females using similar procedures. To measure *C*. *tropicalis* male fertility, we crossed males with *unc-23(v277)* hermaphrodites and scored progeny as either Unc (self) or non-Unc (cross).

### Sperm activation and microscopy

L4 males were placed onto new NGM dishes without hermaphrodites for 48–60 hours. The males were then dissected and sperm released into a droplet of SM buffer (50 mM HEPES, 45 mM NaCl, 25 mM KCl, 1 mM MgCl_2_, 5 mM CaCl_2_, 10 mg/mL PVP, pH 7.0). These sperm were maintained in a chamber constructed by mounting a 22×30 mm glass coverslip onto a glass slide over parallel strips of two-sided sticky tape. Then, 1 mM zinc, 200 μg/mL Pronase, or 1 mg/mL trypsin was poured into the chamber and incubated for 10–15 minutes to activate the sperm. After the treatment, sperm were observed using an Axio Imager M2 microscope (Carl Zeiss) with DIC optics.

For whole worm observations, young adult worms were placed on a 5% agarose pad with a droplet of 1 mM levamisole and viewed with DIC optics. Images were captured using a Zeiss Axiocam digital camera with Zeiss AxioVision software and assembled using Adobe Photoshop.

### Semiquantitative RT-PCR for Fig 4A

Groups of five adult worms were collected and processed as described [57]; two independent samples were used to confirm reproducibility. RNA was extracted as described, and the reverse transcription reaction was performed with MMLV Reverse Transcriptase (ThermoFisher Scientific). The PCR was conducted using HotMaster Taq DNA polymerase (5PRIME); PCR reactions were run for 30 cycles for the *zipt-7*.*1* and *zipt-7*.*2* genes or 24 cycles for the *act-1* gene. Primers are listed in S2 Table.

### Quantitative RT-PCR for Fig 5C

We used the qRT-PCR method described by Davis and colleagues [58], with minor modifications. Mixed-stage populations of animals were cultured for 16 hours on NAMM dishes supplemented with 0 or 40 μM TPEN, seeded with concentrated *Escherichia coli* OP50, and collected by washing. RNA was isolated using TRIzol (Invitrogen), treated with Dnase I, and reverse transcribed with the High Capacity cDNA Reverse Transcription kit (Applied Biosystems). PCR was performed using an Applied Biosystems 7900 thermocycler and iTaq Universal SYBR Green Supermix (Bio-Rad). Primers used to detect *zipt-7*.*1* are in S2 Table.

### RNAi

Double-stranded RNA (dsRNA) was synthesized using T7 RNA polymerase. The templates were amplified from nematode cDNA with PCR primers that contained the T7 promoter (S2 Table), purified with a PCR Purification kit (Qiagen), and transcribed using MegaScript (Ambion). After annealing overnight at 37°C, dsRNA was purified with MegaClear (Ambion). RNAi was performed by injection into hermaphrodites, and the progeny of injected animals were analyzed at the young adult stage, 24 hours after being picked as L4 larvae.

### Fluorescence assays for zinc

To visualize the distribution of zinc, we stained isolated spermatids with 10 μM Zinpyr-1 (Sigma-Aldrich) in divalent cation-free SM for 10 minutes at room temperature. LysoTracker Red and MitoTracker Red (ThermoFisher Scientific) were used at a final concentration of 1 μM to label membranous organelles [59] and mitochondria, respectively. Some spermatids were activated by Pronase after staining with dyes to identify differences in the distribution of zinc between spermatids and spermatozoa. Fluorescent images were obtained using an Olympus Fluoview 1200 confocal microscope.

### Nematode immunocytochemistry

To investigate GFP::ZIPT-7.1 expression and localization, we isolated the male gonad and fixed with 4% paraformaldehyde in SM buffer at 4°C overnight. Fixed samples were permeabilized with 0.5% Triton X-100 in PBS for 1 hour and then blocked with 2% BSA in PBS at room temperature for 6 hours. The primary anti-GFP Mab (Roche, 1:100 dilution) was incubated with samples at 4°C overnight. The secondary antibody was a goat anti-mouse IgG conjugated to horseradish peroxidase. Visualization used 5 minutes of Tyramide signal amplification with Alexa Fluor 488 conjugated to tyramine (ThermoFisher Scientific). Slides were mounted in solution (83 mg/mL mowiol, 25 mg/mL DABCo, 1 mg/mL DAPI in 100 mM Tris buffer, pH 8.0) and images captured using an Olympus Fluoview 1200 confocal microscope.

### Anti-ZIPT-7.1 antibodies

Rabbit polyclonal anti-ZIPT-7.1 antibodies were generated and purified by YenZyme. Two peptides based on the predicted ZIPT-7.1 protein sequence were used as antigens (#68–82, TSHREIQHSRLSTLK, and #138–150, SLSPHDHSHDHHD).

### Plasmid construction

Plasmid pCT15, which was used to express ZIPT-7.1 in cultured cells, was constructed by inserting a 1,182-nucleotide *zipt-7*.*1* cDNA encoding the full-length predicted protein and Kozak sequence into pcDNA3.1(+) (Invitrogen), using restriction sites *Hind*III and *Xba*I. The *zipt-7*.*1* cDNA was produced by performing RT-PCR on a sample of *C*. *elegans* RNA. The plasmid was verified by DNA sequencing.

### Cellular immunocytochemistry

HeLa cells were grown on coverslips, transfected, fixed in 4% formaldehyde for 30 minutes at room temperature, and permeabilized in 0.1% Saponin/2% IgG-free BSA/PBS for 30 minutes. The primary antibodies were polyclonal rabbit antibodies against ZIPT-7.1 used in combination with mouse monoclonal antibodies against Golgi GM130 (1:1,000) (Abcam), Calnexin MAB3126 (1:250) (Millipore), or Lamp2 CD107b (1:200) (Pharmingen). The secondary antibodies were goat anti-rabbit antibodies conjugated with Alexa488 and goat anti-mouse antibodies labelled with Alexa594 (Invitrogen). Cells were mounted in Vectashield/DAPI (Vector Laboratories). Confocal fluorescent images were taken with a 63×/1.4NA planapo objective (Zeiss) on a spinning disc confocal microscope UltraVIEW VoX/Observer.Z1 (PerkinElmer; Zeiss) using Velocity 6.3. Images were exported as 16-bit TIFs and converted into 8-bit RGB TIFs using Fiji ImageJ, and the figures were assembled in Photoshop (Adobe).

### Cell lines and cell culture

HEK293T cells were cultured in Dulbecco’s Modified Eagle Medium (DMEM) (ThermoFisher) supplemented with 10% fetal bovine serum (FBS) (Sigma) and 1× Penicillin/Streptomycin Solution (Corning). HeLa cells were cultured in DMEM supplemented with 2 mM glutamine, 10% FBS, and Penicillin/Streptomycin. All cells were grown in a humidified incubator with 5% CO_2_ at 37°C.

### Zinc uptake assays

Zinc uptake assays were performed as described, with minor modifications [60, 61]. Briefly, HEK293T cells were seeded on Poly-D-lysine–coated 24-well plates (Corning). The next day, the cells were transfected with a plasmid encoding ZIPT-7.1 or pcDNA-3.1(+) (a vector only control) using Lipofectamine 2000 (Invitrogen). After 48 hours, cells were washed once with prewarmed uptake buffer (15 mM HEPES, 100 mM glucose, 150 mM KCl, pH 7.0) and incubated for 15 minutes in prewarmed uptake buffer that contained the radioactive tracer ^65^ZnCl_2_ (PerkinElmer) and varying concentrations of nonradioactive metal ions. Uptake was halted by applying the same volume of ice-cold stop buffer (15 mM HEPES, 100 mM glucose, 150 mM KCl, 1 mM EDTA, pH 7.0). Cells were gently washed with ice-cold stop buffer twice and disassociated with trypsin. The radioactivity that had been incorporated into the cells was then measured with a Beckman LS 6000 Scintillation Counter. In parallel experiments conducted without adding metals, the cells were lysed with lysis buffer (2 mM Tris-HCl, 150 mM NaCl, 1% Triton X-100), and protein levels were measured with the Bio-Rad DC protein assay. ^65^Zn uptake was normalized to total protein measured in this parallel assay. These metals were obtained from Sigma: ZnCl_2_, MgCl_2_, CdCl_2_, FeCl_3_ (Fe^3+^ was reduced to Fe^2+^ with 1 mM sodium ascorbate shortly before the experiment), MnCl_2_, and CaCl_2_. The data shown in Fig 5J–5L are typical of multiple independent experiments.

### Genome editing with TALENs to produce *ctr-zipt-7*.*1* mutants

TALEN mRNAs were designed and produced as described [25] (S3 Table). To create mutants, we injected pairs of TALEN mRNAs into the gonads of young adult hermaphrodites. The F_1_ progeny from a 6- to 30-hour time window were singled to new dishes and raised at 20°C. F_1_ worms that carried new mutations were identified by PCR analysis of the target site (primers in S3 Table), and mutants were confirmed by DNA sequencing.

### Genome editing with CRISPRs to make a *gfp*::*zipt-7*.*1* insertion strain

To generate the GFP::ZIPT-7.1 expression strain, we used CRISPR technology [50] to modify the endogenous locus, so as to encode GFP between amino acids 25 and 26 of ZIPT-7.1. We designed the sgRNA sequence (TCCTTCATGGTGATGCTCGTGG) using the CRISPR design tool (http://crispr.mit.edu). The target site conformed to the sequence G(N19)NGG; the initial G optimizes transcription driven by the U6 promoter, and the NGG (PAM) motif is required for Cas9 activity.

To insert the sgRNA sequence into the CRISPR-Cas9 vector pDD162 (Addgene, #47549), the vector was amplified using primers that had a 15-bp overlap sequence (primers in S2 Table) and Phusion high-fidelity DNA polymerase (New England Biolabs). Next, the PCR products were treated with *Dpn*I to remove the vector template and transformed into TOP10 competent cells. The plasmid was confirmed by DNA sequencing.

To insert *gfp* into the endogenous *zipt-7*.*1* locus, we built a homologous recombination template comprised of left-arm sequence (1,031 bp), GFP deleted-stop-codon sequence with a 6 amino acid linker, and right-arm sequence (1,316 bp). The left- and right-arm sequences were amplified from genomic DNA using Phusion high-fidelity DNA polymerase and primers P1 and P5 (S2 Table) and inserted into the pPD95.77 backbone by In-fusion cloning (ClonTech). Next, the GFP sequence was amplified and inserted between the left and right arms. Positive clones were identified by the PCR, and the final plasmid was confirmed by DNA sequencing.

We injected Cas-9-sgRNA, homologous recombination template, and the selection marker pRF4 (*rol-6*) into N2 hermaphrodites at 50 ng/μL concentration. F_1_ Rol progeny were placed on individual dishes and harvested for PCR analysis after laying eggs for one to two days. We used primers P3 and P6 to identify worms that contained the *gfp* insert, and primers P2, P3, and P4 to screen their progeny for homozygotes (S2 Table). The *gfp* knock-in worms were confirmed by DNA sequencing the PCR product of primers P2 and P4.

### Split-ubiquitin yeast two-hybrid analysis

The split-ubiquitin yeast two-hybrid assay used the DUALmembrane kit (Dualsystems Biotech) per manufacturer's instructions. The *zipt-7*.*1* gene was amplified from *him-5* cDNA using primers Sp24-1Forward and Sp24-1179Reverse (S2 Table). Next, we amplified a truncated portion of *zipt-7*.*1* using primers Sp24-1Forward and C-Sfi-Sp24-1116Reverse, and cloned this fragment into the bait plasmid pBT3-STE. This construct expresses a form of ZIPT-7.1 lacking the last 21 C-terminal amino acids, which includes the final predicted transmembrane domain (S3 Fig). Based on the predicted topology of ZIPT-7.1, this alteration is necessary to localize the ubiquitin tag (which is fused to the C-terminal end of ZIPT-7.1) in the cytoplasm. Details describing the constructs for each prey protein are described elsewhere [62].

The yeast strain NMY51 was transformed with ZIPT-7.1 bait plasmid and selected on synthetic medium lacking leucine. These yeasts were then transformed with prey plasmid and selected on synthetic medium lacking both leucine and tryptophan. Each resulting strain harbored the bait plasmid and one type of prey plasmid; the ability to turn on reporter genes was tested by plating on synthetic medium lacking Trp, Leu, His, and Ade. Yeast cells transformed with bait plasmid and pAI-Alg5, which expresses wild-type Nub (N-terminal ubiquitin), served as a positive control. Yeast cells transformed with bait plasmid and empty prey vectors pPR3-STE or pPR3-N were used as negative controls.

## Supporting information

S1 Fig *zipt-7*.*1* is required for hermaphrodite fertility and sperm activation. **(A)** Values are the total eggs (left) and unfertilized oocytes (right) laid by hermaphrodites over their entire lifetimes. Box and whisker plots show the mean (dotted line), 25th to 75th percentiles (box), and 10th to 90th percentiles (whiskers). Points falling outside of this range are marked individually. All strains contained the *him-5(e1490)* mutation. For wild-type broods *N* = 8, and for *zipt-7*.*1(hc130)* broods *N* = 11. Statistical comparisons used the Mann-Whitney *U* test. (B) DIC images show representative sperm dissected from *XX* hermaphrodites. The *zipt-7*.*1(+)* sperm had pseudopods, indicated by dotted lines, which are indicative of the in vivo activation typically observed in hermaphrodites (left). By contrast, *zipt-7*.*1(*−*)* hermaphrodite sperm were round and did not display extended pseudopods, suggesting these sperm had not activated in vivo (right). DIC, differential interference contrast.
(TIF)

S2 Fig Labile zinc colocalizes with mitochondria and lysosomes in spermatids. (A,B) Photomicrographs of inactive spermatids or mature sperm that had been activated with Pronase. Spermatids were isolated from *him-5(e1490)* males by dissection and stained with Zinpyr-1 to display labile zinc levels, and either LysoTracker to visualize the membranous organelles (A) or MitoTracker to visualize mitochondria (B). The fluorescence photomicrographs display labile zinc levels (left, green), organelles (left center, red), or both dyes (right center, yellow). DIC optics display cell morphology (right). In each set of four pictures, the same two cells are outlined with dotted white lines, based on their DIC images. Scale bars are 5 μm. The yellow color in the overlap images shows that a subset of zinc puncta in spermatids colocalizes with the membranous organelles near the plasma membrane and mitochondria in the interior of the cell. Following activation, the mature sperm extend a pseudopod that does not display labile zinc, membranous organelles, or mitochondria. (C) Comparison of wild-type or *zipt-7*.*1(ok971)* spermatids that were treated with Pronase and stained with Zinpyr-1. Both strains contained the *him-5(e1490)* mutation. Although many *zipt-7*.*1* spermatids failed to activate, some successfully activated, and these did not display labile zinc in the pseudopod. DIC, differential interference contrast.
(TIF)

S3 Fig ZIPT-7.1 localizes to internal cell membranes. (A) Model showing the location of peptides in ZIPT-7.1 used to generate antibodies in rabbits: residues 68–82 (blue) and 138–150 (green). Numbers indicate predicted transmembrane segments. The model shows a predicted membrane topology based on TMpred [63], but other algorithms give somewhat different results regarding the number of transmembrane domains (varies from 6 to 8) and whether there is a signal peptide, so the diagram shows only one of several possibilities. The illustration was generated using Protter [64]. (B, C) HeLa cells were transfected with a plasmid expressing ZIPT-7.1, whole cell lysates were separated by SDS-PAGE, and ZIPT-7.1 protein was detected with the indicated antibodies by western blotting. MW markers in kDa are shown at left. Lanes 1–4 had decreasing amounts of lysate (shown as μL input) from cells expressing ZIPT-7.1 (+), and lane 5 had lysate from untransfected control cells (−). A red arrowhead marks ZIPT-7.1 based on its predicted molecular weight (43.3 kDa) and absence from the control. (D–R) HeLa cells were transfected with a plasmid expressing ZIPT-7.1, fixed in formaldehyde, and double labelled with antibodies against ZIPT-7.1 (68–82) (D, G, green), ZIPT-7.1 (138–150) (J, M, green), or against both ZIPT-7.1 (68–82) and ZIPT-7.1 (138–150) (P, green), and *Cis*-Golgi marker protein GM130 (E, K, red), lysosomal marker protein LMP2 (H, N, red), or ER marker protein calnexin (Q, red). Confocal fluorescence micrographs of representative HeLa cells show that (1) ZIPT-7.1 did not colocalize with the ER marker (R), (2) the epitope recognized by aZIPT-7.1 (68–82) colocalized primarily with the Golgi marker (F) and minimally with the lysosome marker (I), and (3) the epitope recognized by aZIPT-7.1 (138–150) colocalized primarily with the lysosome marker (O) and minimally with the Golgi marker (L). Arrowheads indicate apparent colocalization of ZIPT-7.1 with GM130 or LMP2 (yellow). Bar, 5 μm. ER, endoplasmic reticulum; kDa, kilodalton; MW, molecular weight.
(TIF)

S4 Fig ZIPT-7.1 interacts with SPE-4 but not other sperm activation proteins. Photomicrograph showing the density of yeast colonies at three plating dilutions. The split-ubiquitin yeast two-hybrid study was conducted with ZIPT-7.1 expressed from the bait plasmid (green) and other proteins expressed from the prey plasmid (blue, see Materials and methods for details). The box highlights robust colony growth with SPE-4, indicating a protein–protein interaction. The top four rows are reproduced from Fig 7B and used proteins cloned in the PRN vector. The bottom five rows used proteins cloned in the PRSTE vector.
(TIF)

S1 Table Nomenclature for *C*. *elegans* ZIPT proteins. ZIPT, ZRT- and IRT-like protein transporter.
(DOCX)

S2 Table Primers used in this study. (DOCX)

S3 Table Design of TALENs. TALEN, Transcription activator-like effector nuclease.
(DOCX)

S1 Data Data for Figs 1–7 and S1A Fig. (XLSX)

## Abbreviations

DIC: differential interference contrast
DMEM: Dulbecco’s Modified Eagle Medium
dsRNA: double-stranded RNA
FBS: fetal bovine serum
GFP: green fluorescent protein
LZA: low zinc activation
MSP: major sperm protein
qRT-PCR: quantitative RT-PCR
RNAi: RNA interference
RT-PCR: Reverse transcription polymerase chain reaction
TPEN: N,N,N',N'-tetrakis(2-pyridinylmethyl)-1,2-ethanediamine
ZIPT: ZRT- and IRT-like protein transporter
