# Supplementary figures and images for: The zinc transporter ZIPT-7.1 regulates sperm activation in nematodes
Source: PLoS Biol. 2018 Jun 7;16(6):e2005069. doi: 10.1371/journal.pbio.2005069 (PMC5991658; doi:10.1371/journal.pbio.2005069)

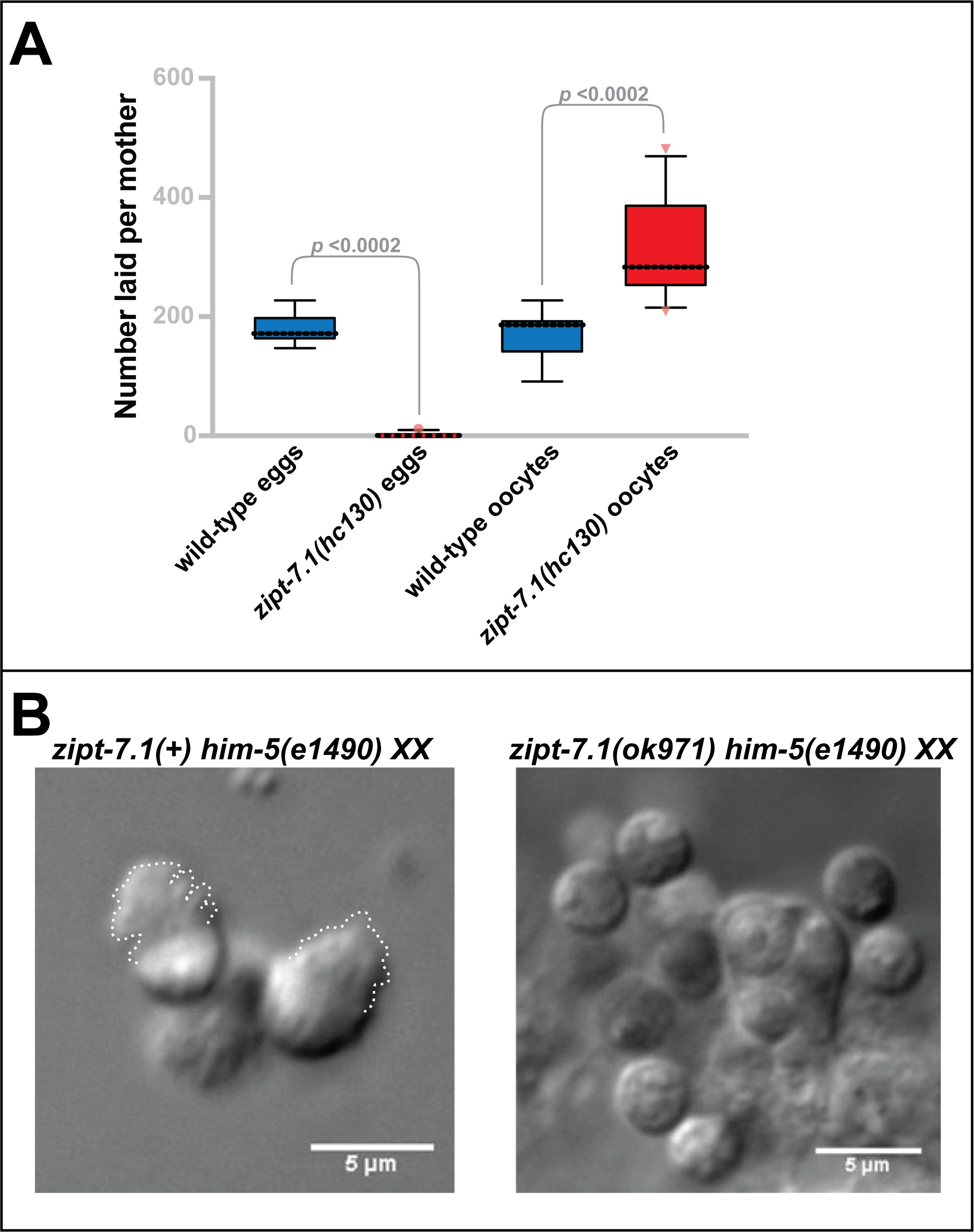

Supplement: S1 Fig — (A) Values are the total eggs (left) and unfertilized oocytes (right) laid by hermaphrodites over their entire lifetimes. Box and whisker plots show the mean (dotted line), 25th to 75th percentiles (box), and 10th to 90th percentiles (whiskers). Points falling outside of this range are marked individually. All strains contained the him-5(e1490) mutation. For wild-type broods N = 8, and for zipt-7.1(hc130) broods N = 11. Statistical comparisons used the Mann-Whitney U test. (B) DIC images show representative sperm dissected from XX hermaphrodites. The zipt-7.1(+) sperm had pseudopods, indicated by dotted lines, which are indicative of the in vivo activation typically observed in hermaphrodites (left). By contrast, zipt-7.1(−) hermaphrodite sperm were round and did not display extended pseudopods, suggesting these sperm had not activated in vivo (right). DIC, differential interference contrast. (TIF) [file pbio.2005069.s001.tif]

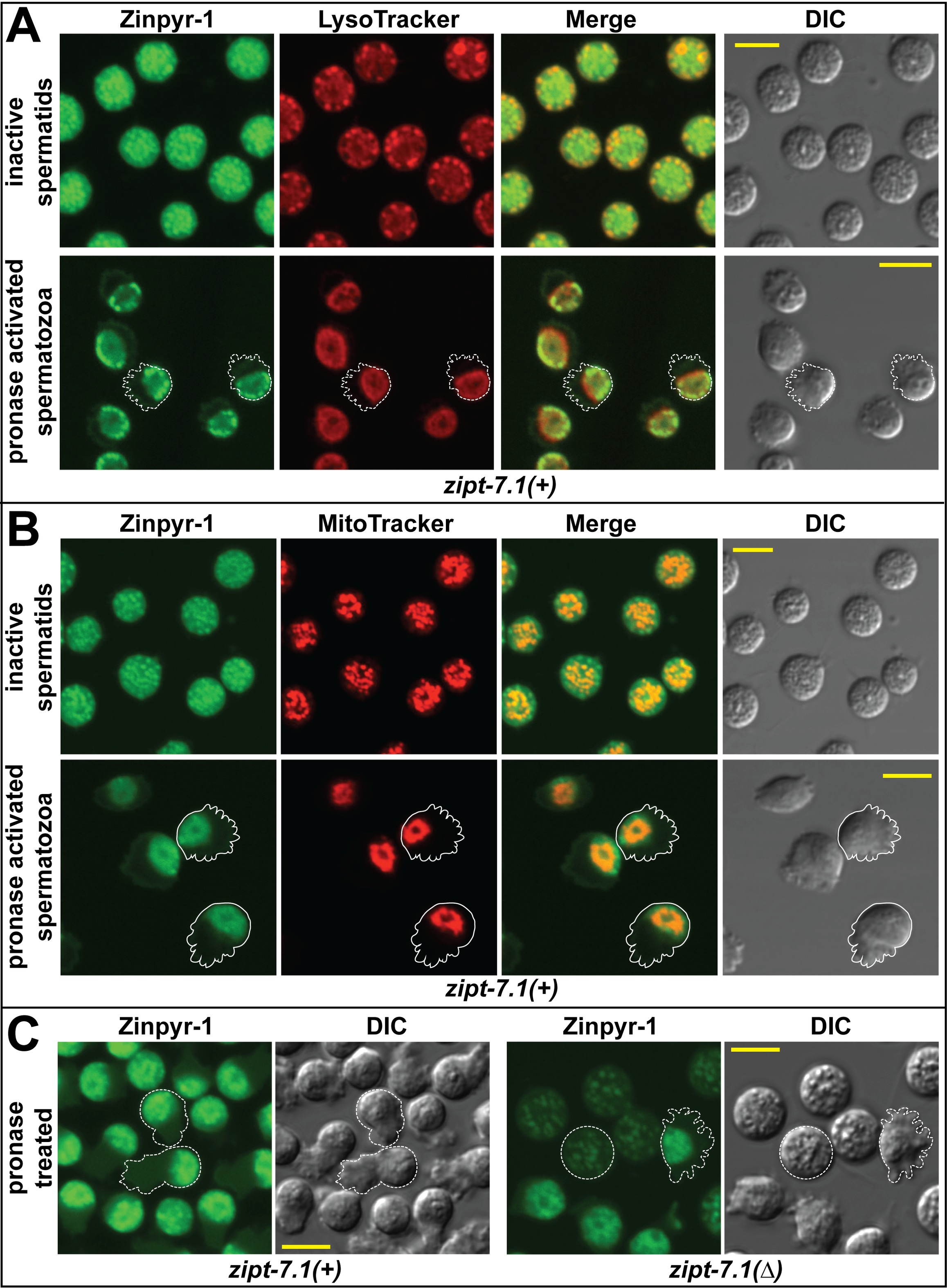

Supplement: S2 Fig — (A,B) Photomicrographs of inactive spermatids or mature sperm that had been activated with Pronase. Spermatids were isolated from him-5(e1490) males by dissection and stained with Zinpyr-1 to display labile zinc levels, and either LysoTracker to visualize the membranous organelles (A) or MitoTracker to visualize mitochondria (B). The fluorescence photomicrographs display labile zinc levels (left, green), organelles (left center, red), or both dyes (right center, yellow). DIC optics display cell morphology (right). In each set of four pictures, the same two cells are outlined with dotted white lines, based on their DIC images. Scale bars are 5 μm. The yellow color in the overlap images shows that a subset of zinc puncta in spermatids colocalizes with the membranous organelles near the plasma membrane and mitochondria in the interior of the cell. Following activation, the mature sperm extend a pseudopod that does not display labile zinc, membranous organelles, or mitochondria. (C) Comparison of wild-type or zipt-7.1(ok971) spermatids that were treated with Pronase and stained with Zinpyr-1. Both strains contained the him-5(e1490) mutation. Although many zipt-7.1 spermatids failed to activate, some successfully activated, and these did not display labile zinc in the pseudopod. DIC, differential interference contrast. (TIF) [file pbio.2005069.s002.tif]

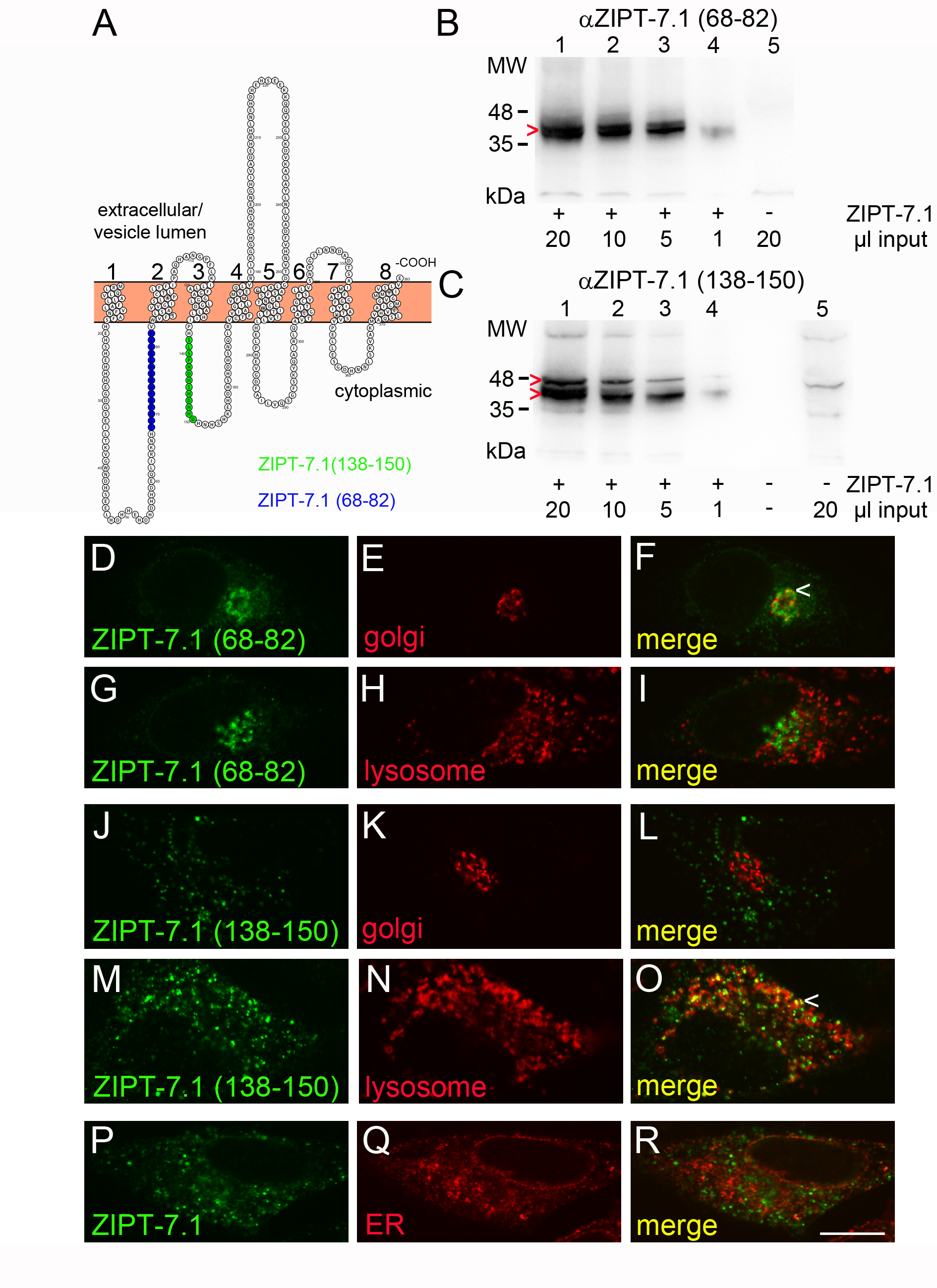

Supplement: S3 Fig — (A) Model showing the location of peptides in ZIPT-7.1 used to generate antibodies in rabbits: residues 68–82 (blue) and 138–150 (green). Numbers indicate predicted transmembrane segments. The model shows a predicted membrane topology based on TMpred [63], but other algorithms give somewhat different results regarding the number of transmembrane domains (varies from 6 to 8) and whether there is a signal peptide, so the diagram shows only one of several possibilities. The illustration was generated using Protter [64]. (B, C) HeLa cells were transfected with a plasmid expressing ZIPT-7.1, whole cell lysates were separated by SDS-PAGE, and ZIPT-7.1 protein was detected with the indicated antibodies by western blotting. MW markers in kDa are shown at left. Lanes 1–4 had decreasing amounts of lysate (shown as μL input) from cells expressing ZIPT-7.1 (+), and lane 5 had lysate from untransfected control cells (−). A red arrowhead marks ZIPT-7.1 based on its predicted molecular weight (43.3 kDa) and absence from the control. (D–R) HeLa cells were transfected with a plasmid expressing ZIPT-7.1, fixed in formaldehyde, and double labelled with antibodies against ZIPT-7.1 (68–82) (D, G, green), ZIPT-7.1 (138–150) (J, M, green), or against both ZIPT-7.1 (68–82) and ZIPT-7.1 (138–150) (P, green), and Cis-Golgi marker protein GM130 (E, K, red), lysosomal marker protein LMP2 (H, N, red), or ER marker protein calnexin (Q, red). Confocal fluorescence micrographs of representative HeLa cells show that (1) ZIPT-7.1 did not colocalize with the ER marker (R), (2) the epitope recognized by aZIPT-7.1 (68–82) colocalized primarily with the Golgi marker (F) and minimally with the lysosome marker (I), and (3) the epitope recognized by aZIPT-7.1 (138–150) colocalized primarily with the lysosome marker (O) and minimally with the Golgi marker (L). Arrowheads indicate apparent colocalization of ZIPT-7.1 with GM130 or LMP2 (yellow). Bar, 5 μm. ER, endoplasmic reticulum; kDa, kilodalton; MW, molec [file pbio.2005069.s003.tif]

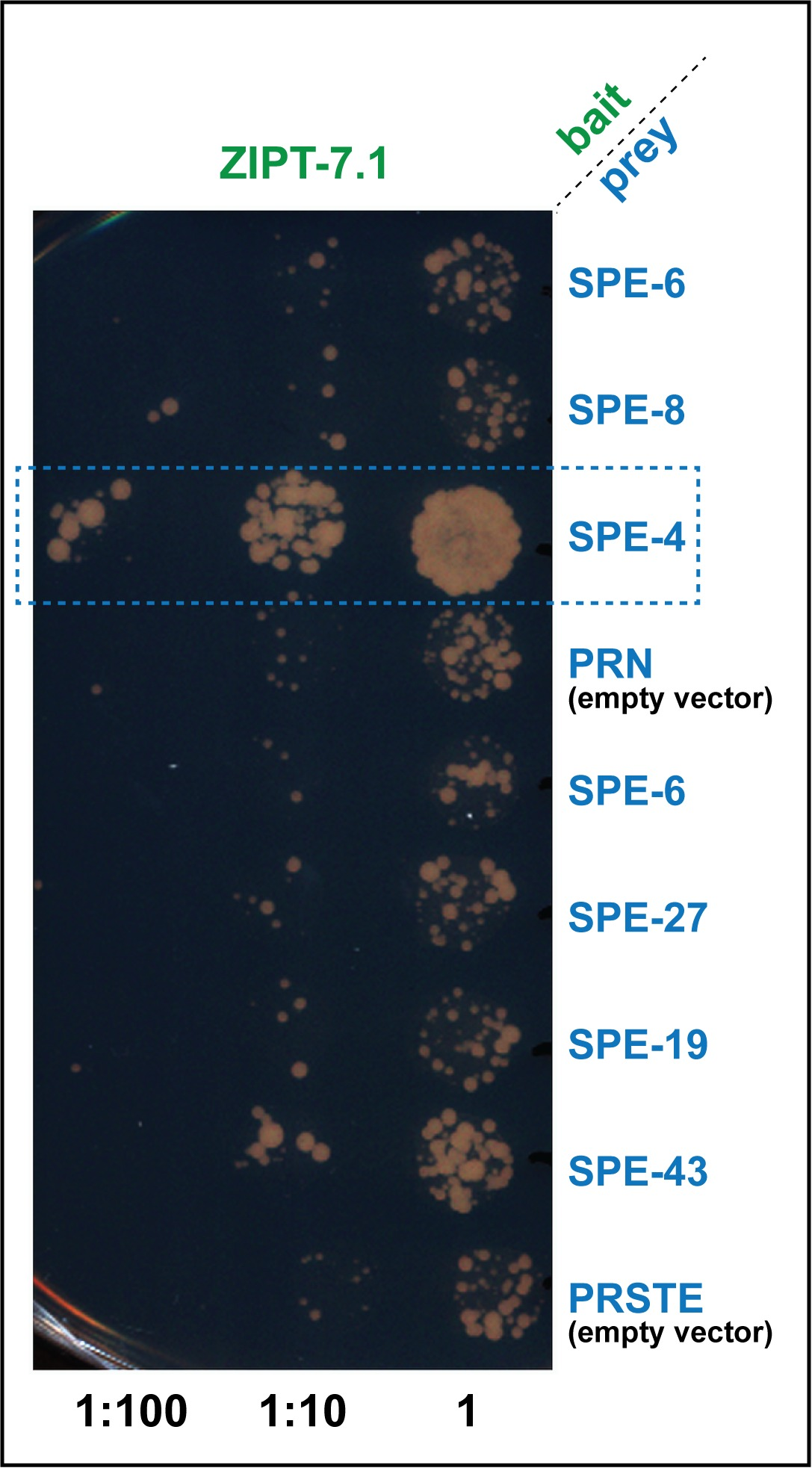

Supplement: S4 Fig — Photomicrograph showing the density of yeast colonies at three plating dilutions. The split-ubiquitin yeast two-hybrid study was conducted with ZIPT-7.1 expressed from the bait plasmid (green) and other proteins expressed from the prey plasmid (blue, see Materials and methods for details). The box highlights robust colony growth with SPE-4, indicating a protein–protein interaction. The top four rows are reproduced from Fig 7B and used proteins cloned in the PRN vector. The bottom five rows used proteins cloned in the PRSTE vector. (TIF) [file pbio.2005069.s004.tif]
