# Supplementary material for: The zinc transporter ZIPT-7.1 regulates sperm activation in nematodes
Source: PLoS Biol. 2018 Jun 7;16(6):e2005069. doi: 10.1371/journal.pbio.2005069 (PMC5991658; doi:10.1371/journal.pbio.2005069)
Supplement: S1 Table — ZIPT, ZRT- and IRT-like protein transporter. (DOCX) [file pbio.2005069.s005.docx]

**S1 Table. Nomenclature for *C. elegans* ZIPT proteins.**

| **Human Ortholog** | ***C. elegans* Protein** | **New *C. elegans* name** |
| --- | --- | --- |
| ZIP1 | F31C3.4 | ZIPT-1 |
| ZIP3 | C18A3.2 | ZIPT-3 |
| ZIP7 | T28F3.3/HKE-4.1 | ZIPT-7.1 |
| ZIP7 | H13N06.5/HKE-4.2 | ZIPT-7.2 |
| ZIP9 | T01D3.5 | ZIPT-9 |
| ZIP11 | F59A3.4 | ZIPT-11 |
| ZIP13 | C14H10.1 | ZIPT-13 |
|  |  |  |
| Perhaps ZIP2 | C06G8.3 | ZIPT-2.1 |
|  | F30B5.7 | ZIPT-2.2 |
|  | Y54G9A.4 | ZIPT-2.3 |
|  | F55F8.9 | ZIPT-2.4 |
|  |  |  |
| Unclear | Y55F3BL.2 | ZIPT-15 |
|  | T11F9.2/TAG-140 | ZIPT-16 |
|  | C30H6.2/TAG-141 | ZIPT-17 |
