# Supplementary material for: The zinc transporter ZIPT-7.1 regulates sperm activation in nematodes
Source: PLoS Biol. 2018 Jun 7;16(6):e2005069. doi: 10.1371/journal.pbio.2005069 (PMC5991658; doi:10.1371/journal.pbio.2005069)
Supplement: S2 Table — (DOCX) [file pbio.2005069.s006.docx]

**S2 Table. Primers used in this study**

| **Description** | **Primer sequence** |
| --- | --- |
| ***Primers used for semi-quantitative RT-PCR*** | |
| *zipt-7.1* rt F | TTCTCCCTTCTTGTCTCAAATC |
| *zipt-7.1* rt R | TACATCATTCCAACACCCATAC |
| *zipt-7.2* rt F | CAACCTCGCTGCTGATTT |
| *zipt-7.2* rt R | GAGCATGGCCTTCTTCTTAG |
| *act-1* rt F | CACCATGTACCCAGGAATTG |
| *act-1* rt R | GAGAGGGAAGCGAGGATAG |
| ***Primers used to make template for RNA interference*** | |
| *zipt-7.1*-T7 F | TAATACGACTCACTATAGGGAGACTCCTTGCACACTTCTCTTC |
| *zipt-7.1*-T7 R | TAATACGACTCACTATAGGGAGAGTTCAGGGATCCAGGATTTG |
| ***Primers used to identify* ctr-zipt-7.1 *mutants following gene editing*** | |
| *Ctr-zipt-7.1* scr F | CCTTTCTTCAATTGTCCTCAATG |
| *Ctr-zipt-7.1*scr R | GCAGAATTCCACTTCCTTCT |
| *Ctr-zipt-7.1*seq F | TGACGCAATGGGTAGAATG |
| *Ctr-zipt-7.1*seq R | TGTGCAGGAATGAAGAAGAG |

| ***Primers used for plasmid constructions*** | |
| --- | --- |
| *Cas9-zipt-7.1* sgRNA F | CTTCATGGTGATGCTCGGTTTTAGAGCTAGAAATAGC |
| *Cas9-zipt-7.1* sgRNAR | AGCATCACCATGAAGGACaagacatctcgcaatagga |
| P1 (Template left arm F) | GTACCGGTAGAAAAAGAGATCACTCTCACCACAGAAG |
| P5 (Template right arm R) | GGAATTCTACGAATGCTCGACCAAACTGACGATGTA |
| 95.77 no GFP linear F | CATTCGTAGAATTCCAACTGAGCG |
| 95.77 no GFP linear R | TTTTTCTACCGGTACCCTCAAGGG |
| Template sgRNA site mutant F | AGTCATGAGCATAGTCACGAGCATCACCATGAAGGA |
| Template sgRNA site mutant R | ACTATGCTCATGACTGAAAGCGTTGAACACTAACGCG |
| GFP-linker F | ATGAGTAAAGGAGAAGAACTTTTCAC |
| GFP-linker R | GTGATCTTCATGGTGATCTTTGTATAGTTCATCCATGCCATG |
| Left-GFP-Right F | CACAGCCACGAGCATATGAGTAAAGGAGAAGAACTTTTCAC |
| Left-GFP-Right R | GTCTCCTTCATGGTGGTGATCTTCATGGTGATCTTTGTATA |
| Left-Right-backbone linear F | CACCATGAAGGAGACGGAAG |
| Left-Right-backbone linear R | ATGCTCGTGGCTGTGTTC |
| ***Primers used to identify* zipt-7.1(ibp18, gfp insertion) *mutants*** | |
| P3 (GFP KI scr F) | CAAAGATGACGGGAACTACAA |
| P6 (GFP KI scr R) | ACCGGGAGATGGAGATTT |
| P2 (GFP KI homo F) | CGTACCCGGTTTCGAATTT |
| P4 (GFP KI homo R) | GAAGAGAAGTGTGCAAGGAG |
| ***Primers used for split ubiquitin assays*** | |
| Sp24-1Forward | ATGCGATTGCAACTTGTCGCCTTG |
| Sp24-1179Reverse | CTCGACCAAACTGACGATGTACATCATTCC |
| C-Sfi-Sp24-1116Reverse | AACTGATTGGCCGAGGCGGCCTTGCTCTGCGCCATTTTTGCCA |
| ***Primers used for quantitative RT-PCR*** | |
| zipt-7.1 qRTF | AGCTGGAAATACTCTTGGATGG |
| zipt-7.1 qRTR | CAGTAACGGCTTGCAAACG |
