# Supplementary material for: The zinc transporter ZIPT-7.1 regulates sperm activation in nematodes
Source: PLoS Biol. 2018 Jun 7;16(6):e2005069. doi: 10.1371/journal.pbio.2005069 (PMC5991658; doi:10.1371/journal.pbio.2005069)
Supplement: S3 Table — TALEN, Transcription activator-like effector nuclease. (DOCX) [file pbio.2005069.s007.docx]

**S3 Table. Design of TALENs**

| **TALEN target sequence used to generate DNA double-strand breaks.** | |
| --- | --- |
| *ctr-zipt-7.1* left arm | **T**GTTTTCTCCAATGAAC |
| Target DNA site | Acagtcaccatcatcac |
| *ctr-zipt-7.1* right arm | TCTGAAGAAGGAAGTGGA**A** |
| **TALEN repeats used to generate mutants** | |
| *ctr-zipt-7.1* left arm | NN NG NG NG NG HD NG HD HD NI NI NG NN NI NI HD |
| *ctr-zipt-7.1* right arm | NG HD HD NI HD NG NG HD HD NG NG HD NG NG HD NI NN NI |
